# Ultrabroad Microwave Absorption Ability and Infrared Stealth Property of Nano-Micro CuS@rGO Lightweight Aerogels

**DOI:** 10.1007/s40820-022-00906-5

**Published:** 2022-08-20

**Authors:** Yue Wu, Yue Zhao, Ming Zhou, Shujuan Tan, Reza Peymanfar, Bagher Aslibeiki, Guangbin Ji

**Affiliations:** 1 College of Materials Science and Technology, Nanjing University of Aeronautics and Astronautics, Nanjing, 210016 People’s Republic of China; 2 Department of Chemical Engineering, Energy Institute of Higher Education, Saveh, Iran; 3 Faculty of Physics, University of Tabriz, Tabriz, 51666-16471 Iran

**Keywords:** Microwave absorption, Ultrabroad bandwidth, Composite aerogel, Radar cross section, Radar-infrared compatible stealth

## Abstract

Developing ultrabroad radar-infrared compatible stealth materials has turned into a research hotspot, which is still a problem to be solved. Herein, the copper sulfide wrapped by reduced graphene oxide to obtain three-dimensional (3D) porous network composite aerogels (CuS@rGO) were synthesized via thermal reduction ways (hydrothermal, ascorbic acid reduction) and freeze-drying strategy. It was discovered that the phase components (rGO and CuS phases) and micro/nano structure (microporous and nanosheet) were well-modified by modulating the additive amounts of CuS and changing the reduction ways, which resulted in the variation of the pore structure, defects, complex permittivity, microwave absorption, radar cross section (RCS) reduction value and infrared (IR) emissivity. Notably, the obtained CuS@rGO aerogels with a single dielectric loss type can achieve an ultrabroad bandwidth of 8.44 GHz at 2.8 mm with the low filler content of 6 wt% by a hydrothermal method. Besides, the composite aerogel via the ascorbic acid reduction realizes the minimum reflection loss (RL_min_) of − 60.3 dB with the lower filler content of 2 wt%. The RCS reduction value can reach 53.3 dB m^2^, which effectively reduces the probability of the target being detected by the radar detector. Furthermore, the laminated porous architecture and multicomponent endowed composite aerogels with thermal insulation and IR stealth versatility. Thus, this work offers a facile method to design and develop porous rGO-based composite aerogel absorbers with radar-IR compatible stealth.

**Supplementary Information:**

The online version contains supplementary material available at 10.1007/s40820-022-00906-5.

## Highlights

- The CuS@rGO composite aerogel can achieve the broad effective absorption bandwidth (EAB) of 8.44 GHz with the filler content of 6 wt%.
- The RL_min_ of CuS@rGO composite aerogel is -55.1 dB and EAB is 7.2 GHz with the filler content of 2 wt% by ascorbic acid thermal reduction. The radar cross-section reduction value of CuS@rGO composite aerogel can reach 53.3 dB m^2^.
- The CuS@rGO composite aerogels possess lightweight, compression and recovery, radar-infrared compatible stealth properties.

**Supplementary Information:**

The online version contains supplementary material available at 10.1007/s40820-022-00906-5.

## Graphical abstract

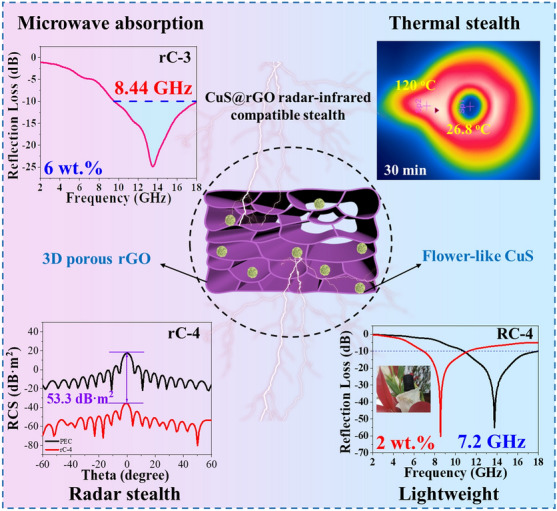

## Introduction

With the fast development of detection technology, stealth materials have attracted extensive attention [1–3]. However, single-waveband stealth materials are hard to satisfy the requirement of harsh environments, and multispectral compatible stealth is becoming the future direction of stealth materials [4–6]. Particularly, with the occurrence of advanced precision-guided weapons and infrared (IR) detectors, designing and exploring the radar-IR compatible stealth materials is of great significance with low IR emissivity and excellent microwave absorbing (MA) ability. Usually, microwave absorbers need low reflectivity and high absorptivity [7–9], while IR stealth materials require high reflectivity and low IR absorptivity [10]. Furthermore, outstanding thermal insulation ability is also required for IR stealth materials according to the Stefan-Boltzmann theory [11]. Thus, it seems to be challenging to integrate IR and radar stealth owing to the thoroughly opposite principles.

To achieve radar-IR compatible stealth, it is of significance to overcome the issue of conflict between IR and radar camouflage material requirements. CuS, a kind of semiconductor transition metal sulfide, has caused broad concern in the IR stealth field owing to the absorbance behavior of local surface plasmon resonance in the near-IR region [12]. At the same time, CuS has also been applied as microwave absorbers due to its exceptional electrical property and unique geometrical micromorphology. For instance, Cui et al. prepared a sandwich-like CuS/Ti_3_C_2_T_*x*_ MXene composites and got the RL_min_ value of − 45.3 dB and the effective absorption bandwidth (EAB) of 5.2 GHz with the filler content of 35 wt% [13]. Quaternary composite of CuS/RGO/PANI/Fe_3_O_4_ was fabricated and the influence of special microstructure on MA capacity was further studied by Wang’s group [14]. The RL_min_ of the products was − 60.2 dB and absorption bandwidth below − 10 dB was up to 7.4 GHz. Liu and his team designed CuS nanoflakes aligned on magnetically decorated graphene via a solvothermal method [15], and found that the different morphologies of nanocomposites showed excellent MA capacity, that was the EAB of 4.5 GHz and RL_min_ value of -54.5 dB. Guan et al. synthesized a series of CuS/ZnS nanocomposites with a 3D hierarchical structure by a hydrothermal method [16]. The obtained nanocomposite possessed the RL_min_ value of − 22.6 dB at 9.7 GHz with the thickness of 3 mm and the EAB of 2.2 GHz (9.2–11.4 GHz). Therefore, CuS-based composites show the application prospects in the field of microwave absorption.

Integrating CuS into thermal-insulating materials is provided a new perspective to design the IR-radar compatible stealth materials. Carbon materials such as carbon nanotubes and graphene have been applied as building blocks to create lightweight and multifunctional microwave absorbers due to their lightweight, conspicuous chemical and mechanical properties, high stability, etc. [17, 18]. Numerous researchers have combined graphene with metallic compounds (ZnO, CeO_2_, MoS_2_, etc.) and magnetic nanoparticles (Ni, Fe, Co, or its alloys) or magnetic compounds (typical ferrites) to fabricate composite powder absorbers that can achieve the integration of dielectric/magnetic loss, and optimize the impedance mismatch owing to the poor impedance matching form single graphene [19, 20]. Although they have achieved excellent MA ability, these composites are hard to meet the other functions for unique applicated environments. Besides, common powder materials also have high filler contents and density. In recent years, aerogels with high porosity (> 95%) and extremely low density (< 0.1 g cm^−3^) have been attractive to researchers [21]. Among them, graphene-based aerogels consisting of interconnected 3D networks of graphene sheets are gained wide attention for their low cost and density, facile synthesis, unique porous structure, and large specific surface area. Moreover, the porous graphene-based aerogels possess the superior thermal-insulating effect for the existence of high porous, air phase, and 3D network structure. The studies on graphene/Ni aerogel [22], CoFe_2_O_4_/N-doped reduced graphene oxide aerogel [23], polyaniline/graphene aerogel [24], and SiC whiskers/reduced graphene oxide aerogel [25] have further confirmed that the composition regulation of graphene-based composite aerogels is conducive to achieving effective absorption bandwidth (EAB) and reducing the filler contents.

Currently, foams and aerogels with porous network structure, high porosity, high specific surface area, such as melamine hybrid foam [26], chitosan-derived carbon aerogels [27], porous carbon@CuS [11], antimony tin oxide/rGO aerogels [28], cobalt ferrite/carbon nanotubes/waterborne polyurethane hybrid aerogels [29], Fe/Fe_2_O_3_@porous carbon composites [30], cellulose-chitosan framework/polyaniline hybrid aerogel [31], rGO/MWCNT-melamine composite [32], organic-inorganic hybrid aerogel [33], and rGO/Fe_3_O_4_ [34], are commonly applied as radar-IR stealth materials. Although the reported carbon-based radar-IR compatible stealth materials can achieve MA performance and thermal/IR stealth, it is difficult to gain a wide EAB (> 8 GHz) and low IR emissivity (< 6.5) with a low filler content (< 5 wt%).

In this work, two kinds of 3D porous CuS@rGO composite aerogels were synthesized by hydrothermal and ascorbic acid thermal reduction methods and subsequent freeze-drying technique. Thanks to the bicomponent synergistic effect and their unique porous architecture, the obtained composite aerogels achieved MA performance and IR stealth ability. By modulating the additive amounts of CuS powders and thermal reduction ways, the porous CuS@rGO aerogels manifested adjustable MA capacity and IR emissivity. Notably, an excellent MA performance of CuS@rGO (30 mg) aerogel with the widest EAB of 8.44 GHz and RL_min_ of − 40.2 dB at an extremely low filler content of merely 6 wt% could be achieved. Besides, the low IR emissivity of 0.6442 was also obtained by adjusting the additive amounts of CuS. Furthermore, the MA and IR stealth mechanisms of CuS@rGO composite aerogels were investigated in detail. This work exploits a novel path in the design and development of radar-IR compatible stealth materials that can work in the today’s complex environment.

## Experimental Section

### Materials

Copper chloride dihydrate (CuCl_2_·2H_2_O), ethylene glycol (EG), thiourea (CH_4_N_2_S), ascorbic acid and anhydrous ethanol (C_2_H_5_OH) were all bought from the Nanjing Chemical Reagent Co., Ltd. Graphite oxide was provided by Suzhou TANFENG Graphene Tech Co., Ltd. (Suzhou, China). All of the chemical reagents were analytically pure and employed without further purification.

### Preparation of CuS Microspheres

The CuS microspheres were prepared via an ordinary solvothermal strategy. CuCl_2_·2H_2_O (6 mmol) was dissolved in 30 mL of EG, which was named solution A that was quickly turned from blue to dark green. CH_4_N_2_S (24 mmol) was dispersed in another 30 mL of EG that was marked as solution *B* at the same time. Then, solution *B* was poured into solution *A*, and continuously stirred for 0.5 h until the solution became transparent. Next, the final solution was transformed into a Teflon-lined autoclave (100 mL) and maintained at 170 °C for 5 h. The products were collected by centrifugation with distilled water and anhydrous ethanol several times. Finally, the products were dried at 60 °C in a vacuum oven.

### Preparation via the Hydrothermal Method

The 3D porous CuS@rGO composite aerogels were synthesized via a hydrothermal method. First, a certain amount of CuS powders (0, 15, 30, 60, and 120 mg) and 120 mg of multilayer graphite oxide were dispersed into distilled water (30 mL) under ultrasonication for 1 h and subsequently stirred for 0.5 h. Then, the dispersions were placed into a Teflon-lined autoclave (50 mL) and lasted at 120 °C for 12 h. Finally, the obtained CuS@rGO composite hydrogels were dialyzed in anhydrous ethanol/distilled water solution with a volume ratio of 1:9 for 48 h and then freeze-drying at − 50 °C for 48 h to obtain CuS@rGO composite aerogels. The composite aerogels were marked as rC-1, rC-2, rC-3, rC-4, and rC-5.

### Preparation via the Ascorbic Acid Reduction Method

The 3D porous CuS@rGO composite aerogels were synthesized via the ascorbic acid reduction method. First, a certain amount of CuS powders (0, 10, 20, 30, and 40 mg), 80 mg of multilayer graphite oxide and 1.2 g ascorbic acid were dispersed into distilled water (20 mL) under the ultrasonication treatment for 1 h and stirred for 0.5 h. Then, the dispersions were poured into a custom silicone mold (25 mL) at 95 °C for 12 h. Finally, the obtained CuS@rGO composite hydrogels were dialyzed in anhydrous ethanol/distilled water solution with a volume ratio of 1:9 for 48 h and then freeze-drying at − 50 °C for 48 h to obtain CuS@rGO composite aerogels. The composite aerogels were labeled as RC-1, RC-2, RC-3, RC-4, and RC-5.

### Characterization

The composition and crystal structure of CuS@rGO aerogels were investigated by X-ray diffraction (XRD, Bruker D8 ADVANCE, equipped with Cu-K*α* radiation). X-ray photoelectron spectroscopy (XPS) was carried out on a Kratos AXIS Ultra spectrometer with the Al K*α* X-rays as the excitation source. The micromorphology was characterized by a Hitachi S4800 field emission scanning electron microscope (SEM) and a Talos F200X transmission electron microscopy (TEM) equipped with energy dispersive spectrum (EDS).

### Microwave Absorption Measurements

The EM parameters of complex permeability ($\mu_{r} = \mu^{\prime}{-} \, j\mu^{\prime\prime}$) and complex permittivity ($\varepsilon_{r} = \varepsilon^{\prime}{-} \, j\varepsilon^{\prime\prime}$) were measured by the vector network analyzer (VNA, Agilent PNA N5244A) adopting the coaxial line method. The rC aerogels (6 wt%) were mixed with 94 wt% paraffin, and RC aerogels (1 and 2 wt%) respectively mixed with 99 and 98 wt% paraffin, and then pressed into a toroidal ring of the inner diameter of 3.04 mm and out diameter of 7.00 mm.

### Computer Simulation Technology

Computer simulation technology (CST) studio Suite 2018 was applied to simulate the RCS values of as-prepared CuS@rGO composite aerogels under open boundary conditions. The simulation model consisted of the perfect electric conductor (PEC) layer with a thickness of 1.0 mm at the bottom and an absorbing layer with a thickness of 2.0 mm on the top. The dimension of length was equal to the width of 200 mm. Then, the created model was placed on the *x*O*y* plane, and the linear polarized plane EMW was added with the incidence direction on *Z*-axis positive to negative, and the electric polarization was along the *X*-axis. In addition, the far-field monitor frequency was set as 15.7 GHz. The RCS values could be computed as follows [35]:

1 $$ \sigma = 10{\text{log}}\left( {\frac{4\pi S}{{\lambda^{2} }}\left| {\frac{{E_{{\text{S}}} }}{{E_{{\text{i}}} }}} \right|} \right)^{2} $$

where *λ* and *S* are the wavelength of incident wave and area of the simulation model, *E*_i_ and *E*_s_ are the intensity of electric field of the incident and scatted EMWs, respectively.

### IR Stealth Measurement

The IR-2 dual-band IR emissivity meter was used to test the IR emissivity in the waveband of 3 ~ 5 and 8 ~ 14 μm. Thermal IR imaging digital images were recorded by TVS-2000 MK with a heating platform, and the temperature was set as 120 °C.

## Results and Discussion

### Preparation and Reduction Mechanism

**Fig. 1:**
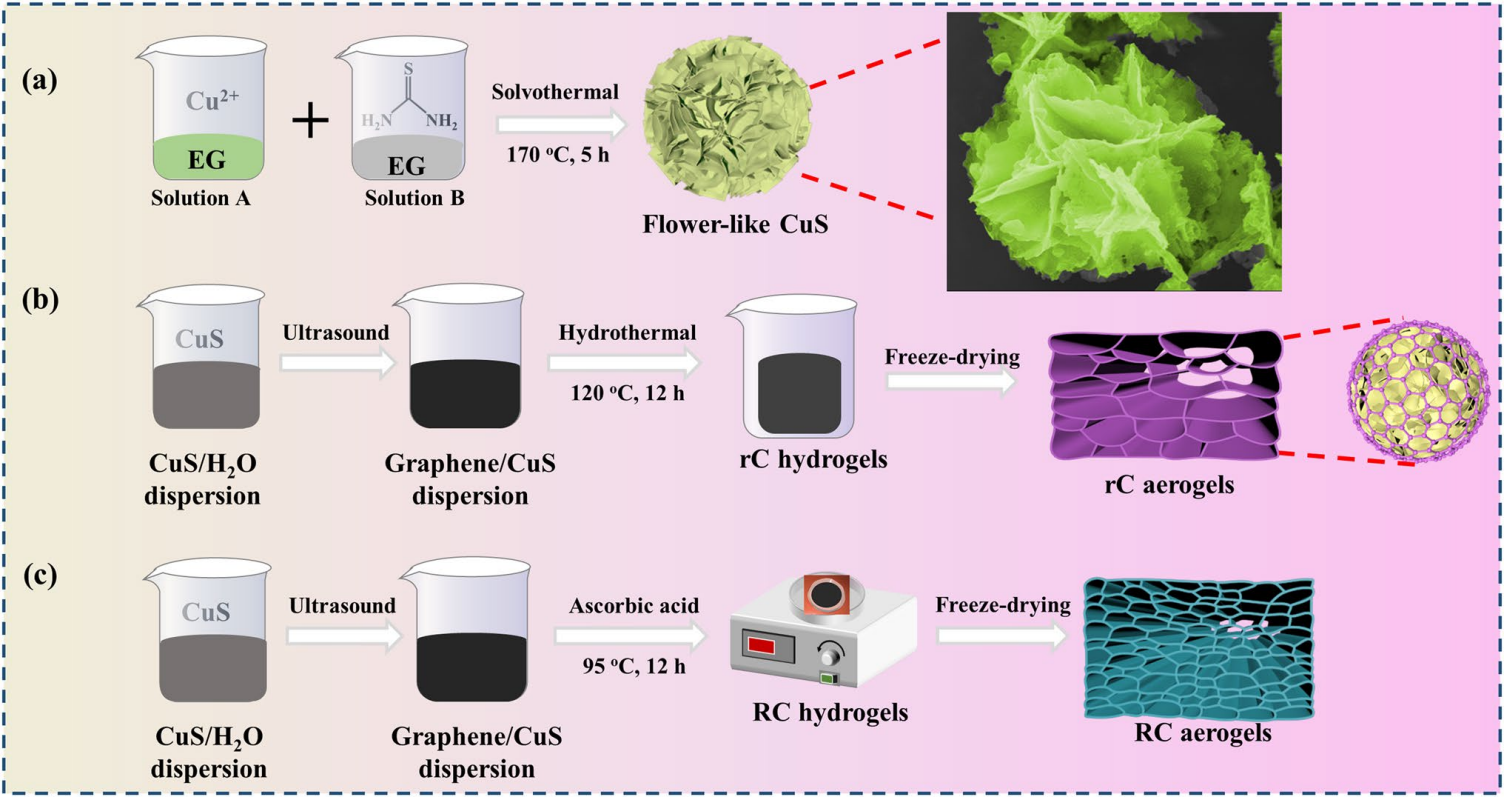
Schematic diagram of preparation processes of **a** flower-like CuS microspheres, and **b, c** CuS@rGO composite aerogels through a hydrothermal method (**b**), and via the ascorbic acid thermal reduction (**c**)

The synthetic processes of CuS@rGO composite aerogels are depicted in Fig. 1. The first step is to fabricate CuS flower-like microspheres via a solvothermal method in Fig. 1a. Then, the 3D porous CuS@rGO composite aerogels were fabricated through complexing CuS in graphene/deionized water dispersion and combining with freeze-drying technique. Hydrothermal (Fig. 1b) and ascorbic acid reduction (Fig. 1c) methods were employed for the preparation of CuS@rGO composite hydrogels, and the freeze-drying technique was applied to obtain the corresponding aerogels with 3D porous architecture. The reduction processes of hydrothermal method can be illustrated in Fig. S1a–b [36]. The carboxyl functional groups can be reduced through a hydrothermal method. As depicted in Fig. S1a, the decarboxylation reaction is accompanied by the production of carbon dioxide. The deoxidation processes of epoxide groups to form a carbon-carbon double bond can be divided into two steps (Fig. S1b). The first step is that the ring of epoxide groups is opened in the existence of formic acid by the acid-catalyzed reaction to produce alcohol in the decarboxylation reaction. The nucleophilic reagent or strong bases can attack the ternary ring of epoxide groups and then relieve the strain energy. Under the circumstances, the hydride ions of formic acid work as nucleophiles at the hydrothermal reaction temperature. First, the epoxide groups are protonated, which activates them to attack the nucleophile. Then, the carbocation is formed that is attacked by hydride ions from formic acid, and the ring is opened to generate alcohol. The second step refers to the dehydration reaction of alcohol to carbon-carbon double bonds with the help of an acidic medium. The -OH (weak leaving groups) needs the protonation reaction to transform it to H_2_O which is easy to leave. A carbocation is formed by water loss, and the water then absorbs the protons to generate carbon-carbon double bonds in rGO. The reduction mechanisms for rGO under the action of ascorbic acid are depicted in Fig. S1c [37]. The carboxyl, epoxy, carbonyl and hydroxyl groups are existed on the surfaces or at the edge of the graphene oxide (GO) sheet. The ascorbic acid can liberate two protons to obtain dehydroascorbic acid, while the protons usually possess a strong affinity with the oxygen-containing groups that can react to form water molecules during the reduction of GO to rGO. At the same time, a number of the neighboring carbon atoms will be taken away as the oxygen-containing functional groups are removed, which can cause vacancy defects in the rGO. Due to the difference in reduction strategies, it can be inferred that the structure of CuS@rGO composite aerogels is also different. Thus, we have further measured the physical parameters of rC composite aerogels. It can be found that the as-prepared aerogels have a few differences in size, including the length, radius and even the mass weight (Table S1). The density of rC composite aerogels is approximate 0.01 g cm^−3^, and is increased with the additive amounts of CuS. The results are that the pure rGO aerogel possesses the lowest density of 0.0110 g cm^−3^, while the rC-5 has the largest density of 0.0160 g cm^−3^.

**Fig. 2:**
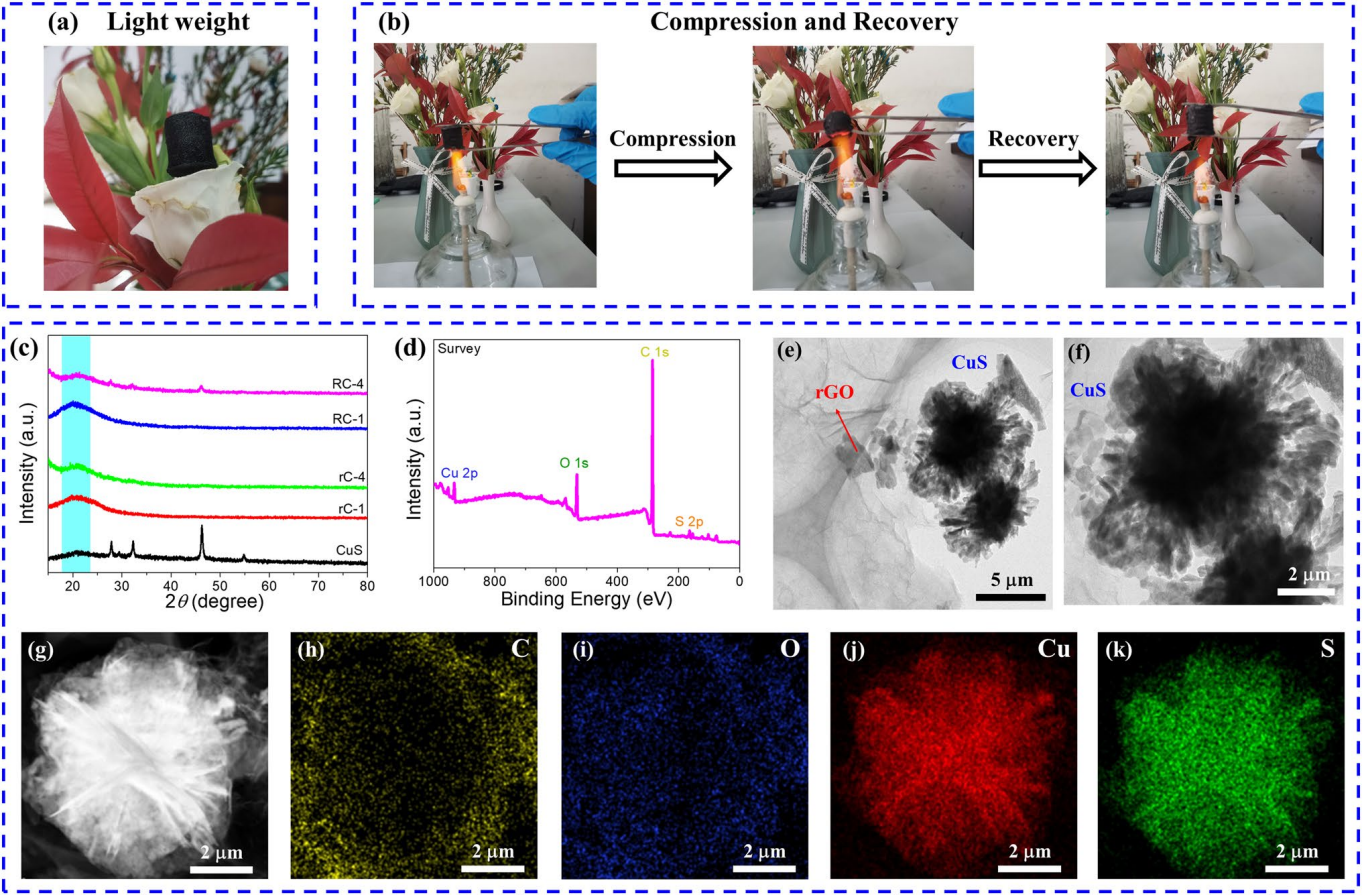
CuS@rGO aerogel characteristics of **a** light weight, **b** compression and recovery. **c** XRD patterns of CuS, rGO, rC-4, RGO and RC-4. **d** XPS full spectrum of rC-4. **e–f** TEM images, and **g–k** EDS mapping images

To confirm the characteristic of lightweight, it is observed that the CuS@rGO composite aerogel can stand on the petals without damaging them at all, demonstrating excellent lightweight feature (Fig. 2a). Besides, the aerogel is observed to express good thermal insulation when placed over the flame of the alcohol lamp. When the aerogel is further compressed with tweezers, it can be well compressed. While the tweezers are released, it can return to its original shape in Fig. 2b, indicating its good compression and recovery characteristic.

The crystalline structure of the prepared CuS@rGO composite aerogels is characterized through XRD analysis. In Fig. 2c, the diffraction peaks at 54.8°, 46.4°, 32.3°, 29.4°, and 27.8° are ascribed to the (108), (110), (103), (102), and (101) crystal planes of CuS (JCPDS No.06–0464) [13]. The rC-1 and RC-1 samples show a broad peak corresponding to the (002) plane of rGO. Besides, the peak intensity becomes weaker with the addition of CuS, and the peak intensity of rGO is too strong, resulting in the relatively weak intensity of CuS. The chemical valence state and surface composition of rC-3 aerogel were measured through XPS. The full spectrum depicted in Fig. 2d confirms the occurrence of S, O, C, and Cu elements that is consistence with the composition of aerogel. From Fig. S2a, the C 1*s* spectrum shows three peaks at 288.9, 285.5, and 284.6 eV, which are assigned to the O–C = O, C–OH, and C–C/C = C bonds, severally [38]. Figure S2b is the Cu 2*p* high-resolution spectrum with two typical peaks at 932.0 and 952.3 eV, corresponding to the Cu 2*p*_3/2_ and Cu 2*p*_1/2_ orbitals of S–Cu bonds [13]. From Fig. S2c, the S 2*p* spectrum can be divided into three peaks, i.e., S–C (168.3 eV), S 2*p*_1/2_ (163.6 eV), and S 2*p*_3/2_ (162.0 eV) [38]. For the O 1*s* spectrum illustrated in Fig. S2d, the obvious peaks at 532.8 and 531.9 eV are indexed to the –OH and lattice oxygen, respectively [38]. The above XPS results further verify the high purity of CuS@rGO composite aerogel.

The morphology and microstructure of CuS and CuS@rGO are observed by SEM. Figure S2e shows a hierarchical flower-like structure of CuS with an around diameter of 5 μm. From Fig. S2f–j, the rC composite aerogels present a typical 3D porous structure composed of overlapping neighboring rCO sheets. Furthermore, the surface of the rGO sheet occurs some holes marked as white boxes. The CuS was wrapped by the rGO sheet when the additive amounts of CuS powders reached 15 mg. In addition, the surface of rGO becomes rougher compared with rC-1 (pure rGO aerogel), which may be the formation of interfaces between CuS and rGO that is conducive to attenuating the incident EMWs. From Fig. S2p, it is more evident that the CuS microspheres are wrapped by rGO sheet from RC-5 (marked by a red dotted box). Interestingly, the rC-3 possesses a larger porous structure than that of other aerogels. The geometrical structure of CuS and rGO of rC-4 was further investigated by the TEM. As depicted in Fig. 2e–f, the rGO and CuS can be easily distinguished from TEM images. The flower-like CuS structure was assembled by 2D nanoflakes, and there are many voids between the interwoven CuS nanosheets. Besides, the rGO exhibits sparse lamellar structure duo to the almost transparent nature of rGO in the CuS@rGO composite aerogel. From Fig. 2g–k, the EDS mapping images of rC-4 demonstrate that the Cu and S elements are chiefly distributed on the CuS microsphere. In addition, C and O elements are distributed throughout the region, indicating the structure of CuS wrapped by rGO sheets. All of these results can well distinguish and see rGO from CuS.

### Microwave Absorption Performance

EM parameters of CuS@rGO composite aerogels synthesized by two different reduction strategies are investigated to deduce the effects of the defects and porous structure on MA performance. The EM parameters and reflection loss of CuS@rGO composite aerogels by hydrothermal reduction and ascorbic acid reduction two methods are calculated as follows [39, 40]:

2 $$ {\text{RL}} = 20{\text{lg}}\left| {\frac{{Z_{{{\text{in}}}} - Z_{0} }}{{Z_{{{\text{in}}}} + Z_{0} }}} \right| $$

3 $$ Z_{{{\text{in}}}} = Z_{0} \sqrt {\frac{{\mu_{{\text{r}}} }}{{\varepsilon_{{\text{r}}} }}} {\text{tanh}}\left( {j\frac{{2\pi fd\sqrt {\mu_{{\text{r}}} \varepsilon_{{\text{r}}} } }}{c}} \right) $$

**Fig. 3:**
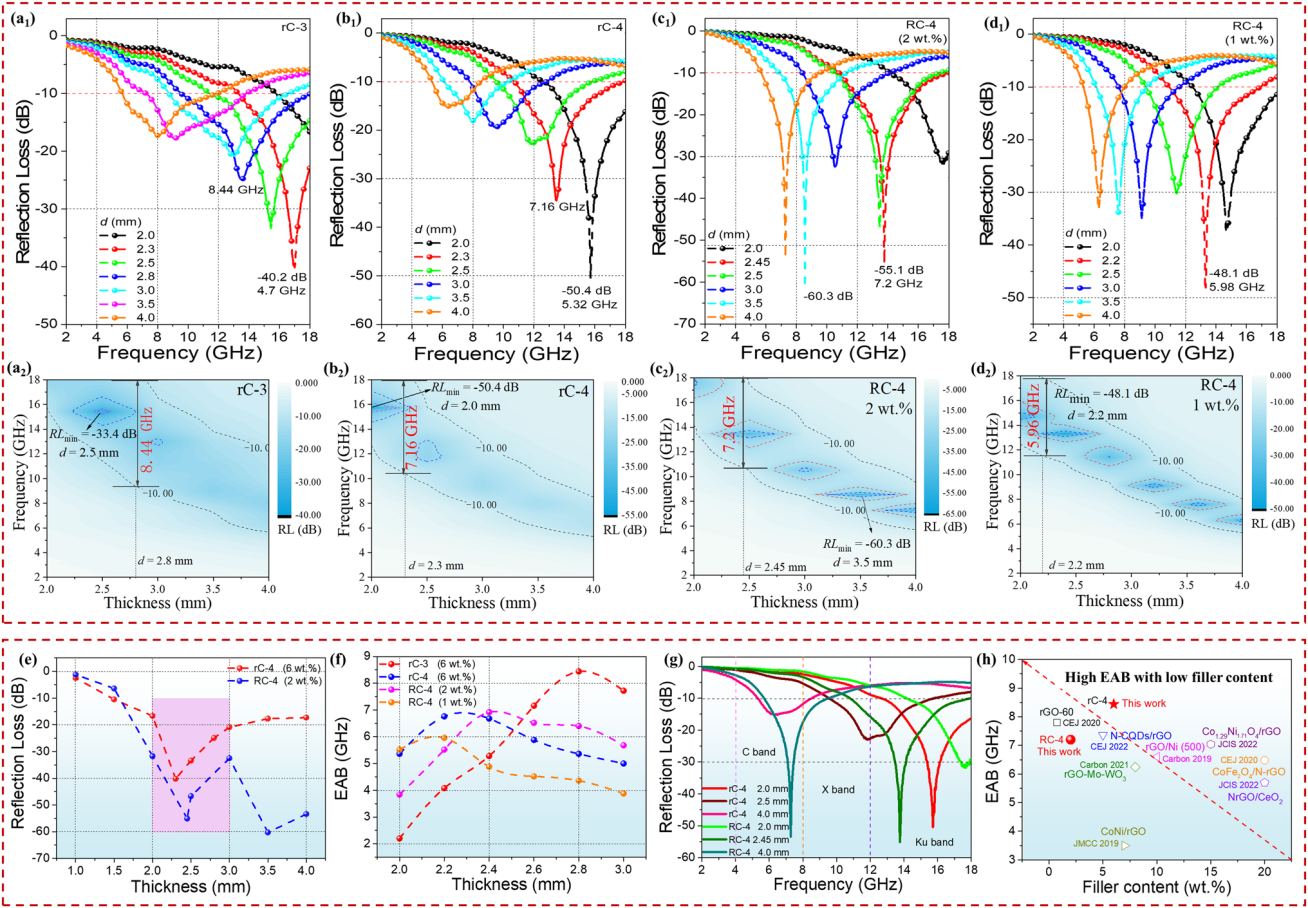
RL curves: **a**_**1**_ rC-3, **b**_**1**_ rC-4, **c**_**1**_ RC-4 (2 wt%) and **d**_**1**_ RC-4 (1 wt%). 2D RL contour maps of **a**_**2**_ rC-3, **b**_**2**_ rC-4, **c**_**2**_ RC-4 (2 wt%) and **d**_**2**_ RC-4 (1 wt%). **e** RL_min_ and **f** EAB at different thickness of rC-4 and RC-4. **g** Selected RL-f curves at various frequency wavebands. **h** Comparison of MA performance considering the EAB and filler contents with reported rGO-based composite aerogels

Herein the physical parameters of *Z*_in_, *Z*_0_, *c*, *f*, *d*, *μ*_r_ and *ε*_r_ represent the input impedance, free space impedance, speed of light, frequency, matching thickness, relative complex permeability and relative complex permittivity, respectively. As depicted in Fig. S3b_1_–b_5_, the *RL*_min_ values of rC aerogels show a trend of increasing first and then declining, that is the RL_min_ values of − 12.3 (2.0 mm), − 16.1 (2.0 mm), − 40.2 (2.3 mm), − 50.4 (2.0 mm), and − 38.4 (3.0 mm) dB, respectively. It is worth noting that the complexing with CuS microspheres is beneficial to improving MA capacity. As depicted in Fig. 3a1–a2, the rC-3 can achieve the RL_min_ of − 40.2 dB and a narrow EAB of 4.7 GHz at 2.0 mm. Furthermore, the broadest EAB is up to 8.44 GHz at 2.8 mm. When the additive content of CuS is 60 mg, the rC-4 obtains the EAB of 7.16 GHz at 2.3 mm and the RL_min_ of − 50.4 dB at 2.0 mm in Fig. 3b1–b2. Interestingly, the RL_min_ values show a shift to low frequency as the thicknesses increase.

The EM parameters include the $\varepsilon^{\prime}$, $\varepsilon^{\prime\prime}$, $\mu^{\prime}$ and $\mu^{\prime\prime}$. The $\mu^{\prime}$ and $\varepsilon^{\prime}$ denote the storage ability of magnetic and electric energy, while $\mu^{\prime\prime}$ and $\varepsilon^{\prime\prime}$ denote the dissipation capacity of magnetic and electric energy, respectively [41]. Owning to the rGO and CuS@rGO without magnetic components ($\mu^{\prime\prime} = 0$ and $\mu^{\prime} = 1$), we merely pay attention on the *ε*_r_ and dielectric loss tangent (tan*δ*_e_). From Fig. S4, the dielectric constants ($\varepsilon^{\prime}$ and $\varepsilon^{\prime\prime}$) descend as the frequency goes up, indicating an obvious frequency dispersion effect that is conducive to attenuating incident EMWs. In addition, with the increase in additive amounts of CuS, the $\varepsilon^{\prime}$ and $\varepsilon^{\prime\prime}$ generally present a decreasing trend. The tan*δ*_e_ of rC aerogels with the order of rC-1 > rC-2 > rC-3 > rC-4 > rC-5 is depicted in Fig. S4c.

Besides, the effects of additive contents of CuS on EM parameters and MA performance of RC composite aerogels via the ascorbic acid reduction strategy with the lower filler content of 2 wt% are also investigated in Fig. S5–S6, Tables S2 and S3. The RL_min_ values are − 32.0 (4.0 mm), − 16.8 (2.5 mm), − 21.5 (2.5 mm), − 60.3 (3.5 mm), and − 16.2 (2.5 mm) dB, respectively. In general, the RC-4 possesses the optimal MA behavior considering the low thickness, strong absorption, and broad bandwidth, *i.e.*, the *RL*_min_ of -55.1 dB and the EAB of 7.2 GHz can be achieved under 2.45 mm. Furthermore, a lower RL_min_ value is − 60.3 dB at 3.5 mm as shown in Fig. 3c1–c2.

From Fig. S6a, the RC-1 has the largest $\varepsilon^{\prime}$ values than that of other RC aerogels, and the range of *ε*′ values for other aerogels is small. The $\varepsilon^{\prime\prime}$ curves of RC aerogels show a familiar downward trend with multiple polarization peaks in 6–18 GHz (Fig. S6b), manifesting the existence of conduction loss and polarization loss. Figure S6c displays the frequency-dependent curves of tan*δ*_e_, which implies that the RC-4 has relatively stronger dielectric loss capacity and the RC aerogels occur polarization peak in high frequency of 11–15 GHz.

Furthermore, the RC composite aerogels with the lower filler content of 1 wt% are studied in Fig. S7. It is seen that the RC composite aerogels show an enhanced MA capacity than pure rGO aerogel (RC-1). Figure S7f more intuitively observed that the absolute values of RL_min_ (|RL_min_|) enhance first and then decline, and RC-4 has the biggest |RL_min_| of 63.5 dB. It is interesting that by changing filler content, the final result of RC-4 has the optimal reflection loss.

The Cole-Cole curves of CuS@rGO aerogel were investigated to further elucidate the polarization relaxation processes. Based on the Debye theory, the $\varepsilon^{\prime}$ and $\varepsilon^{\prime\prime}$ are described as follows:

4 $$ \varepsilon^{\prime} = \varepsilon_{\infty } + \frac{{\varepsilon_{s} - \varepsilon_{\infty } }}{{1 + \left( {2\pi f} \right)^{2} \tau^{2} }} $$

5 $$ \varepsilon^{\prime\prime} = \frac{{2\pi f\tau \left( {\varepsilon_{s} - \varepsilon_{\infty } } \right)}}{{1 + \left( {2\pi f} \right)^{2} \tau^{2} }} $$

Based on the above equations, the correlation between $\varepsilon^{\prime}$ and $\varepsilon^{\prime\prime}$ could be calculated [42, 43]:

6 $$ \left( {\varepsilon^{\prime} - \frac{{\varepsilon_{s} - \varepsilon_{\infty } }}{2}} \right) + \left( {\varepsilon ^{\prime\prime}} \right)^{2} = \left( {\frac{{\varepsilon_{s} + \varepsilon_{\infty } }}{2}} \right)^{2} $$

Herein *ε*_∞_, *ε*_s_, and *τ* are relative complex permittivity at infinite frequency limit, static permittivity, and relaxation time, respectively. Therefore, the curve of $\varepsilon^{\prime\prime}$ *vs* $\varepsilon^{\prime}$ should be a semicircle, called the Cole-Cole semicircle. Generally, each semicircle is on behalf of one Debye relaxation process. From Fig. S4d–h, the curves of all rC aerogels are made up of distorted semicircles and straight tails. The distorted semicircle may be ascribed to polarization relaxation like dipole polarization and interfacial polarization, while the straight line in tail is relevant to conduction loss. It can be discovered that all rC aerogels have at least two semicircles. From Fig. S6d–h, all RC aerogels also have at least two semicircles, indicating the polarization relaxation loss. Compared with rC aerogels, the conduction loss of RC aerogels is much lower from the tail straight. The polarization loss of CuS@rGO aerogels primarily comes from the following aspects. On the one hand, complexing CuS with rGO can be considered as a “capacitor-like” structure that leads to the inhomogeneous distribution and accumulation of free electrons at the heterogeneous interface, enhancing the interfacial polarization to attenuate incident EMWs. On the other hand, CuS, a p-type semiconductor, has ample Cu vacancies, which can result in the unbalance of charges located at the defect sites and then induces dipole polarization. In addition, the –COOH, –OH, etc. on the surface or edge of rGO can also cause dipole polarization.

To compare the effect of reduction way on MA performance, the *RL* and EAB of RC-4 (1 wt%), RC-4 (2 wt%), rC-4 and rC-3 are drawn in Fig. 3e–g. Figure 3e depicts the RL_min_ values of rC-4, RC-4 (2 wt%) at 1.0–4.0 mm. The RC-4 (2 wt%) possesses overall lower RL_min_ values than rC-4. In addition to RL_min_, EAB also should be taken into consideration. From Fig. 3f, RC-4 (1 wt%) has the smallest EAB at 2.4–3.0 mm, and rC-3 reaches the highest EAB at 2.6–3.0 mm. As presented in Fig. 3g, the RL curves of the selected thickness for rC-4 and RC-4 (2 wt%) can occur in different frequency wavebands (C band, X band, and Ku band). The performance comparison about EAB and filler content of this work to other reported rGO-based aerogels has been given in Fig. 3h [23, 44–50]. Most of reported works had higher filler contents or smaller EAB. However, this work can realize the wider EAB and the lower filler content simultaneously.

**Fig. 4:**
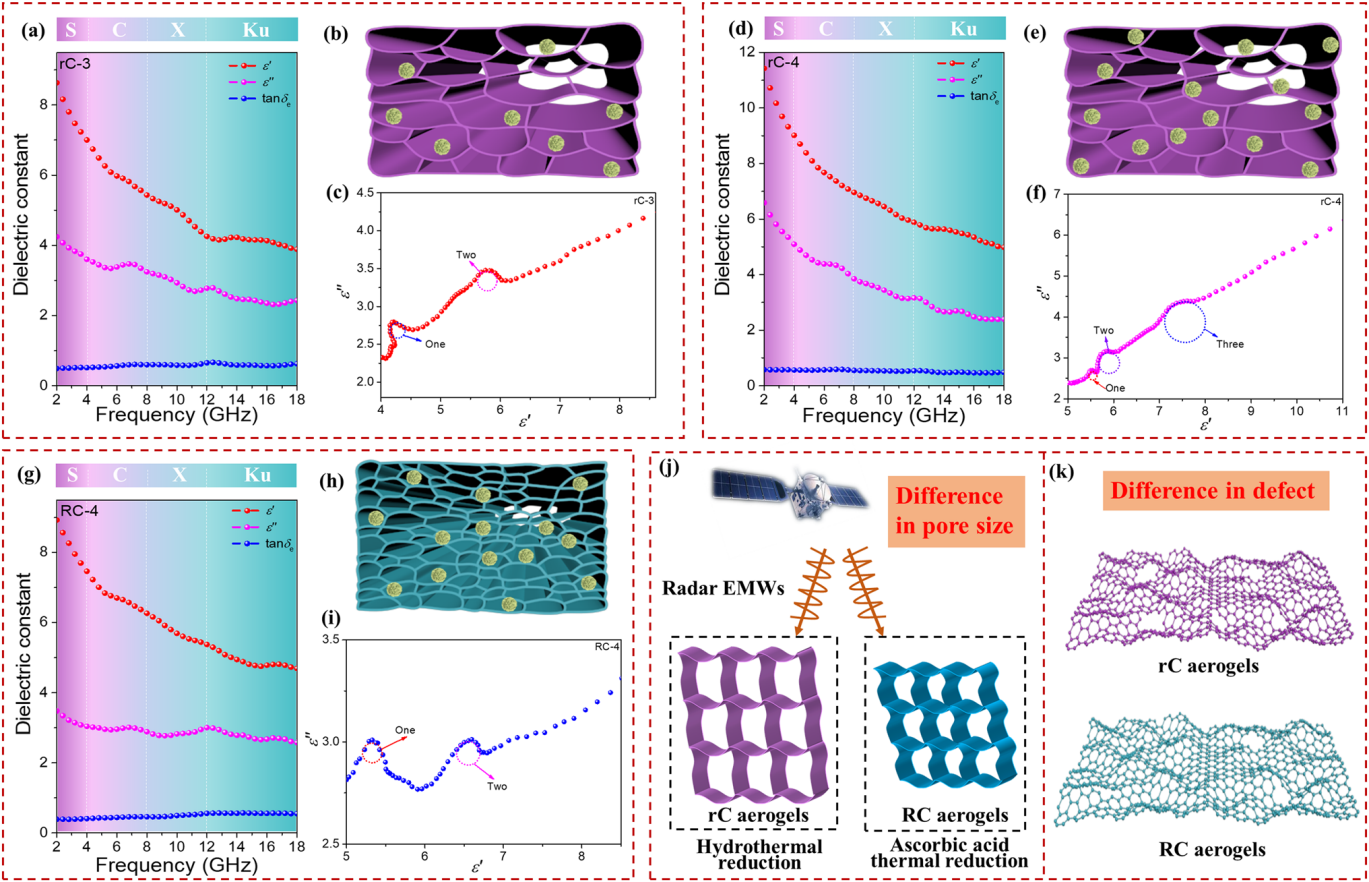
$\varepsilon^{\prime}$, $\varepsilon^{\prime\prime}$, tan*δ*_e_ ~ *f* curves: **a** rC-3, **d** rC-4, and **g** RC-4. Structure diagram: **b** rC-3, **e** rC-4, and **h** RC-4. Cole–Cole curves: **c** rC-3, **f** rC-4, and **i** RC-4. Structure difference of rC and RC composite aerogels in **j** pore size and **k** number of defects

According to the structure of rC composite aerogels (rC-3 and rC-4) and RC-4, the EM parameters and dielectric loss have been further explored in detail. As depicted in Fig. 4a, d, g, the rC-4 has the largest average dielectric constant ($\varepsilon^{\prime}$ and $\varepsilon^{\prime\prime}$), implying the stronger dielectric loss behavior. Due to the difference in additive amounts of CuS and reduction methods, the CuS@rGO composite aerogels display the various structures in Fig. 4b, e, h. Compared with rC-3, rC-4 has a higher content of CuS, which is beneficial to forming the more interfacial polarization. As for rC-4 and RC-4, rGO in rC-4 is reduced at 120 °C, while the RC-4 at 95 °C. Therefore, it is deduced that more defects could be formed in rC-4 than RC-4. Besides, the pore diameter of rC-4 is much larger than RC-4 according to the SEM results, which is more help to attenuate the EMWs. From Cole-Cole curves in Fig. 4c, f, i, the upward tails of rC composite aerogels become longer, suggesting the enhanced conduction loss. So, the structure difference of CuS@rGO composite aerogels with two various reduction methods is presented in Fig. 4j–k. The hydrothermal strategy with the higher temperature can generate more defects and form larger pores than that of the ascorbic acid reduction method.

Usually, attenuation constant (*α*) and impedance matching have a decisive impact on MA capability. The *α* denotes the dissipation capacity of EMWs, which is described as follows [51–53].

7 $$ \alpha = (\sqrt 2 \pi f/c \times \sqrt {\left( {\varepsilon^{\prime\prime}\mu^{\prime\prime} - \varepsilon^{\prime}\mu^{\prime}} \right) + \sqrt {\left( {\varepsilon^{\prime\prime}\mu^{\prime\prime} - \varepsilon^{\prime}\mu^{\prime}} \right)^{2} + \left( {\varepsilon^{\prime\prime}\mu^{\prime} + \varepsilon ^{\prime}\mu ^{\prime\prime}} \right)^{2} } } $$

The larger $\varepsilon^{\prime\prime}$ values can lead to the improved *α* values from Eq. (7) for the $\mu^{\prime} = 1$ and $\mu^{\prime\prime} = 0$. The *α* curves of rC aerogels are shown in Fig. S4i, which keep an escalating tendency at 2–18 GHz. The *α* values with the order of rC-5 < rC-3 < rC-4 < rC-2 < rC-1 reveal that the introduction of low dielectric component CuS would reduce the *α* values. From Fig. S6j, RC aerogels demonstrate the same variation as the frequency increases, while the order of *α* values is RC-2 < RC-5 < RC-3 < RC-4 < RC-1. Since the RC-4 possesses relatively attenuation capacity among composite aerogels, leading to superior MA behavior.

In addition to attenuation loss, another factor, impedance matching (*Z*) also can affect MA performance. Impedance matching is on behalf of the EMWs entering into the absorbents, which can be accessed as follows [54].

8 $$ Z = Z_{{{\text{in}}}} /Z_{0} = \sqrt {\frac{{\mu_{{\text{r}}} }}{{\varepsilon_{{\text{r}}} }}} {\text{tanh}}\left( {j\frac{2\pi fd}{c}\sqrt {\varepsilon_{{\text{r}}} \mu_{{\text{r}}} } } \right) $$

Generally, the optimal impedance matching needs that the *Z* is equal to or close to 1, that is, the input impedance equal to free space impedance (*Z*_in_ = *Z*_0_). As illustrated in Fig. S4j–n, it can be discovered that the |*Z*_in_/*Z*_0_| of rC-1 and rC-2 are much lower than 1, indicating poor impedance matching, and other rC samples are much closer to 1, which is accordance with the reflection loss results that they possess better MA performance than the other two samples. Figure S4o further draws the impedance matching curves of rC aerogels at the thickness of 2.0 mm, which shows the rC-4 is closest to 1 compared with other samples. For RC aerogels, the RC-1 and RC-4 are pretty close to 1 in Fig. S6k–o, manifesting their good absorbing performance (Figs. S5b_1_–d_1_ and S5b_4_–d_4_). The superior performance may be owing to the more defects and functional groups (Fig. S6p).

According to the above results, the *RL*_min_ absorption peaks shift to the low frequency with increasing thicknesses, which can use the explanation of *λ*/4 cancellation theory [55, 56].

9 $$ t_{m} = \frac{nc}{{4f_{m} \sqrt {\varepsilon_{r} \mu_{r} } }}\left( {n = 1,3,5, \ldots } \right) $$

**Fig. 5:**
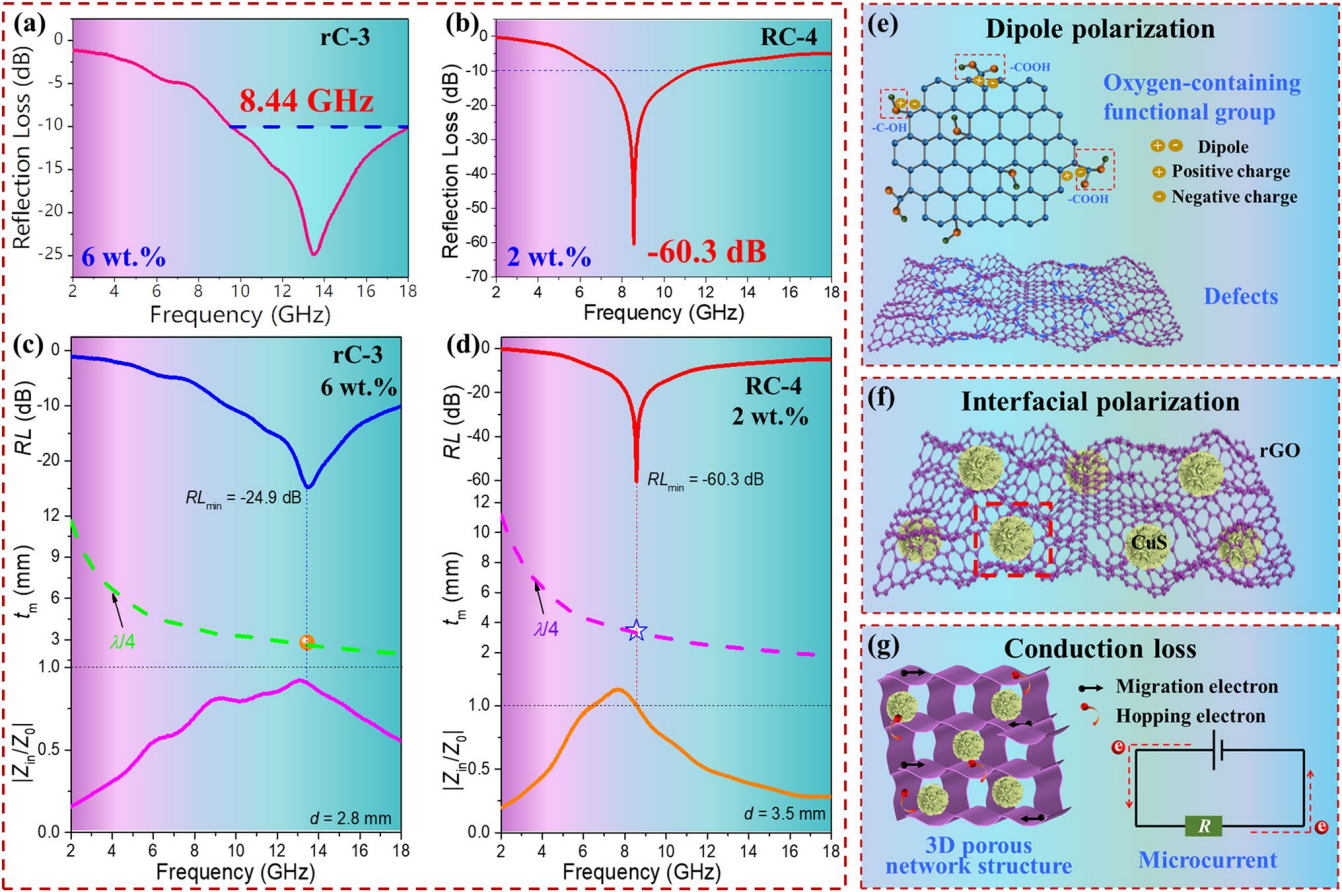
**a** RL ~ *f* curve of rC-3 with the broadest EAB at 2.8 mm. **b** RL ~ *f* curve of RC-4 with the RL_min_ at 3.5 mm. RL, *t*_m_ and |*Z*_in_/*Z*_0_| curves: **c** rC-3 and **d** RC-4. Possible MA mechanism of CuS@rGO composite aerogels: **e** dipole polarization, **f** interfacial polarization and **g** conduction loss

From Fig. 5c-d, compared with rC-3, RC-4 shows the perfect matching point as the RL_min_ is achieved at 8.56 GHz at 3.5 mm that the impedance match is just at 1. Therefore, the RC-4 can satisfy the *λ*/4 wavelength model and perfect impedance matching at the same time, which is conducive to the formation of RL_min_. Besides, the RL, *t*_m_ and |*Z*_in_/*Z*_0_| curves of rC-4 and RC-4 composite aerogels are given in Fig. S8. It is clear that all *t*_m_^exp^ (experimental *t*_m_) values fall perfectly on the *λ*/4 curve, which suggests that the *λ*/4 cancellation model plays a leading role in the relationship between *t*_m_ and *f*_m_.

Based on the discussion of composition, structure and performance, the EMW absorbing mechanism of CuS@rGO is demonstrated in Fig. 5e–g. Firstly, the complex of low dielectric CuS can optimize the impedance matching of pure rGO aerogel. rGO with microporous structure can availably reduce the permittivity for the incorporation of the high-volume fraction of air ($\varepsilon_{r} = 1$), which is helpful to improve impedance matching. The effective permittivity (*ε*_eff_) can be described based on the Maxwell-Garnett model [57, 58].

10 $$ \varepsilon_{{{\text{eff}}}}^{{{\text{MG}}}} = \left[ {\frac{{\left( {\varepsilon_{2} + 2\varepsilon_{1} } \right) + 2p\left( {\varepsilon_{2} - \varepsilon_{1} } \right)}}{{\left( {\varepsilon_{2} + 2\varepsilon_{1} } \right) - p\left( {\varepsilon_{2} - \varepsilon_{1} } \right)}}} \right]\varepsilon_{1} $$

Herein $\varepsilon_{2} ,\varepsilon_{1}$ and *p* are the permittivity of the air phase and solid phase, and the volume fraction of air phase in the porous structure. Typically, the incident EMWs are uninterested in the hole lower than the wavelength, so the micropore and nanopore can act as the effective medium to reduce the $\varepsilon_{eff}$ value for the existence of air. Secondly, the surface or edge of rGO has defects and functional groups, which can induce the formation of dipole polarization [59]. Thirdly, the combination of CuS micro-flower with rGO aerogel can promote the generation of multiple heterogeneous interfaces like CuS/rGO, rGO/paraffin, and CuS/paraffin, causing the stronger interfacial polarization than pure CuS or rGO aerogel [60]. Finally, the interconnected conductive network constructed by rGO sheet can form microcurrents by means of electron migration and hopping, endowing CuS@rGO composite aerogel with excellent conduction loss [61, 62]. As a result, it can be concluded that the CuS@rGO composite aerogels can achieve excellent MA performance due to the unique merits of lightweight, low filler content, compression and recovery, wide absorption bandwidth and strong absorption, which integrates the “thin, light, wide and strong” properties of absorbers.

### Microwave Dissipation Capacity Evaluated by RCS through CST Simulation

**Fig. 6:**
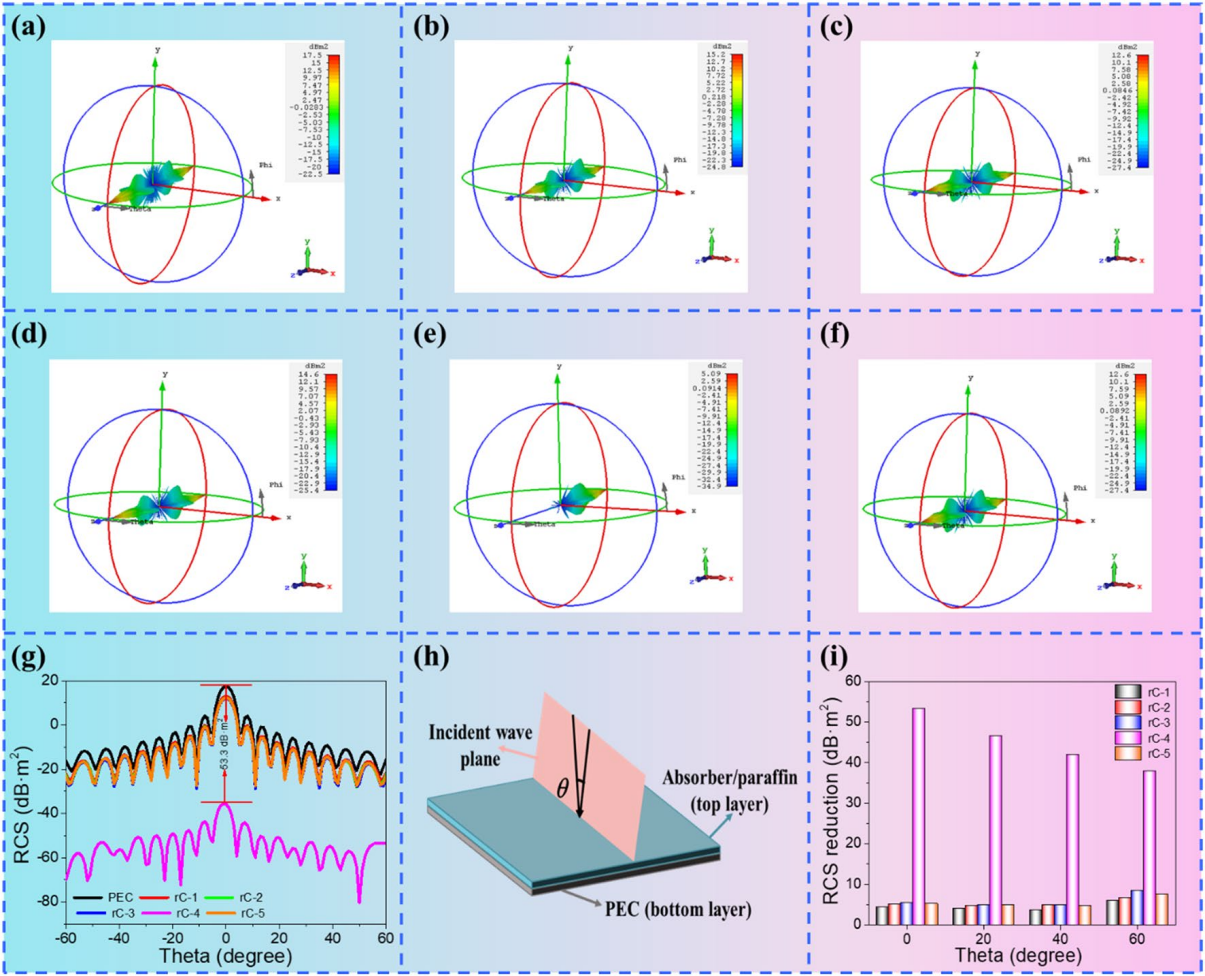
3D radar wave scattering signals of **a** PEC, **b** rC-1, **c** rC-2, **d** rC-3, **e** rC-4 and **f** rC-5. **g** RCS simulated curves of PEC and RC composite aerogels. **h** Schematic diagram of CST simulation. **i** RCS reduction values of RC composite aerogels at the scanning angles of 0°, 20°, 40° and 60^°^

Microwave dissipation capacity of rC composite aerogels in the far-field condition is assessed by the RCS values of rC aerogels covered with the PEC model that are calculated by CST simulation. Figure 6a–f depicts the 3D radar wave scattering signals of PEC and rC aerogels. It is distinct that the rC-4 covered with PEC displays the weakest scattering intensity than other rC aerogels and PEC model, suggesting that the rC-4 possesses the lowest RCS. The detailed RCS value in the − 60° < θ < 60° angle range are presented in Fig. 6g. The PEC has the biggest RCS values, manifesting that rC aerogels can reduce the radar scattering intensities of the pure PEC plate. Besides, RCS value of PEC larger than 0 at 0° is owing to the interference between the reflected EMW and the incident EMW that is perpendicular to the absorber (Fig. 6h). RCS reduction values are further calculated in Fig. 6i. All five samples realize the reduced RCS values compared with the simulated PEC modes, and rC-4 exhibits the highest RCS reduction values at each primary angle. It is up to the maximum value of 53.3 dB m^2^, which is in accord with the minimum reflection loss of rC-4. These results confirmed that with the synergistic effect of dipole polarization, interfacial polarization, conduction loss, and unique porous structure, the EM energy can be effectively dissipated, and the radar scattering intensities are reduced at the same time.

### IR Stealth Performance

To satisfy the demand for radar-IR compatible stealth, the as-prepared CuS@rGO composite aerogels with excellent thermal insulation performance due to the unique porous structure are also necessary in addition to the superior MA ability. The IR radiation will be emitted from the target when the temperature is above absolute zero, which can be detected by the IR detector. Besides, once the target has a high contrast with the background IR radiation, it will be exposed. Reducing the IR radiation energy is the main strategy to achieve IR stealth, originating from the Stefan-Boltzmann equation [63].

11 $$ E\left( T \right) = \int\limits_{0}^{\infty } {\varepsilon \left( {\lambda ,T} \right)c_{1} \lambda ^{{ - 5}} \left[ {\exp \left( {\frac{{c_{2} }}{{\lambda T}}} \right) - 1} \right]^{{ - 1}} d\lambda  = \varepsilon \left( T \right)\sigma T^{4} }  $$

**Fig. 7:**
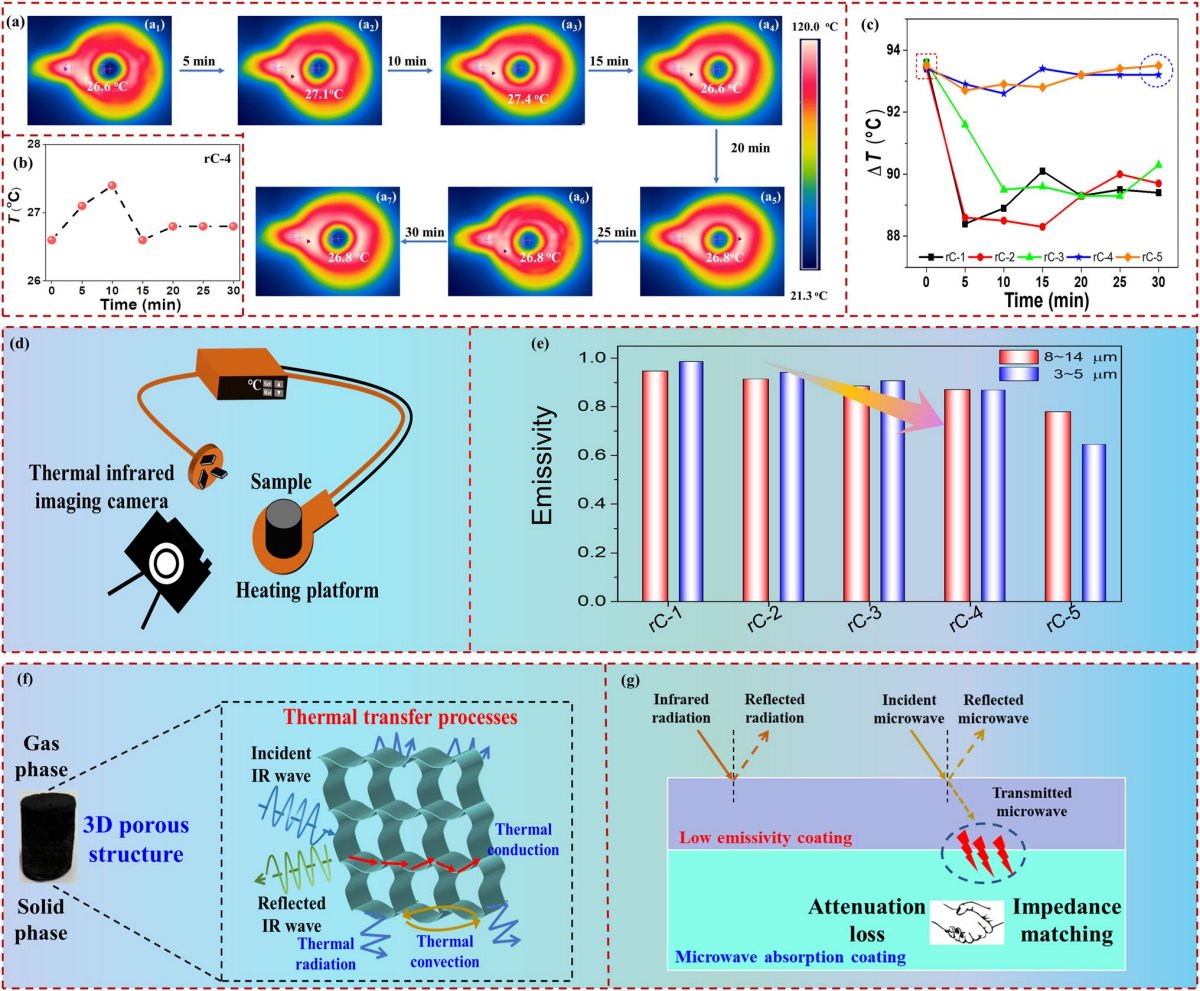
**a** Thermal IR images of rC-4 at different heating times. **b** Surface temperature curve of rC-4. **c** Difference between heating temperature and surface temperature of rC aerogels. **d** Schematic diagram of IR thermal imaging test. **e** IR emissivity of rC composite aerogels at 3 ~ 5 and 8 ~ 14 m. **f** Thermal transfer processes of porous CuS@rGO composite aerogels. **g** Schematic diagram of radar-IR compatible stealth

Herein *E*, *ε*, *T* and *σ* mean IR radiation energy, IR emissivity, surface temperature and Stefan-Boltzmann constant, *c*_1_ and *c*_2_ represent the first and second radiation constant, respectively. Superior thermal stealth can protect targets from detection in the military field. Thus, the IR stealth performance of CuS@rGO composite aerogels was studied by a thermal IR camera. Besides, the IR emissivity is also characterized at 3–5 and 8–14 m via IR-2 Emissometer. The thermal IR images of rC-4 at 10-min intervals are depicted in Fig. 7a. The rC-4 aerogel is placed in the center of a circular heating platform (Fig. 7d), and the heating temperature is set to 120 °C. The surface temperature of rC-4 is 26.6 °C at the beginning. From Fig. 7b, it is interesting that the surface temperature will go up at a tiny temperature difference (surface temperature and maximum temperature, Δ*T* < 0.8 °C), and then it can maintain almost its original temperature after 30 min heating, indicating its stable thermal stealth capability. The other CuS@rGO aerogels are tested with the same condition and their results are depicted in Figs. S9–S12 and Table S4. It can be more intuitively seen from Figs. S13 and 7c that the Δ*T* is decreasing, and rC-5, in particular, has almost no temperature difference, suggesting that the surface temperature of rC composite aerogels is much closer to the beginning temperature after 30 min heating with the increase in CuS content. These results further confirm that complexing low-emissivity CuS with 3D porous rGO aerogel is conducive to thermal stealth ability. The abundant air with lower thermal conductivity can take the place of solid phase with higher thermal conductivity. Besides, 3D aerogels endow with a low density and porous structure, and a large number of pores inside hinder the heat transfer. The existence of CuS microspheres also obstruct the heat transfer between rGO sheets. Therefore, the CuS@rGO composite aerogels have excellent thermal insulation performance. Furthermore, low IR emissivity is another way to realize IR stealth. The IR radiation energy can be reduced by modulating the emissivity with unchanged surface temperature. There are currently two atmospheric window regions of 3 ~ 5 and 8 ~ 14 m adopted by IR detectors. As presented in Fig. 7e and Table S2, the IR emissivity of rC composite aerogels shows a downward trend on the IR waveband of both 3 ~ 5 and 8 ~ 14 m, which is consistence with the results of thermal IR images. Besides, the emissivity at 3 ~ 5 m is much lower than 8 ~ 14 m. The possible IR stealth mechanism is summarized in Fig. 7f. The forms of thermal transfer consist of thermal radiation, thermal conduction and thermal convection, which all occur in CuS@rGO aerogels. Owing to the low density of porous aerogels, the gas-phase components can reduce the thermal conduction for their low thermal conductivity. Moreover, the 3D network structure is conducive to prolonging the thermal transfer path and reducing the thermal conduction in the solid phase, leading to a perfect insulation performance. Figure 7g shows the ideal double-layer radar-IR stealth coating. The EMWs can pass through the IR stealth layer, and enter the MA layer, then be dissipated. Impedance matching is one of the most significant factors in minimizing the radar reflectivity of IR stealth coating.

## Conclusions

In this work, we developed an effective composite-structure-performance strategy to enhance MA performance and reduce IR emissivity. Two types of CuS@rGO composite aerogels were successfully fabricated via hydrothermal reduction and ascorbic acid thermal reduction. The reduction mechanisms involved the decarboxylation process, dehydroxylation process, and deoxidation process of epoxy groups, which could lead to the defects. In addition, adjacent graphene sheets wrapped by numerous tiny CuS are stacked with each other to form a 3D porous structure during the thermal reduction process. The porous structure and defects could be modulated by the thermal reduction and additive amounts of CuS. Because of the balanced attenuation capability and impedance matching, the as-prepared CuS@rGO aerogels depicted impressive microwave absorbing performance. The CuS@rGO aerogels achieved the broadest EAB of 8.44 GHz (2.8 mm) with the additive amount of 30 mg. The samples realized the RL_min_ of − 50.4 dB (2.0 mm) with the additive amount of 60 mg through the hydrothermal reduction method under the filler content of 6 wt%. Besides, the CuS@rGO aerogel (RC-4) could achieve the EAB of 7.2 GHz and RL_min_ of − 55.1 dB at 2.45 mm with the filler content of 2 wt%, in addition, the RL_min_ of − 48.1 dB and EAB of 5.96 GHz could be obtained at 2.2 mm with the lowest filler content of 1 wt%. The CST simulated results also demonstrated that the CuS@rGO composite aerogels could effectively reduce the radar scattering intensity. Furthermore, thermal IR images and IR emissivity could confirm that the GuS@rGO composite aerogels had the ability to reduce the surface temperature and IR emissivity. Thus, these results will lead to the development of radar-IR compatible stealth materials composed of carbon-based aerogels, which can make them a considerable application prospect in a harsh military environment.

## Supplementary Information

Below is the link to the electronic supplementary material.

Supplementary file1 (PDF 2343 KB)
