# Supplementary material for: Ultrabroad Microwave Absorption Ability and Infrared Stealth Property of Nano-Micro CuS@rGO Lightweight Aerogels
Source: Nanomicro Lett. 2022 Aug 20;14:171. doi: 10.1007/s40820-022-00906-5 (PMC9392679; doi:10.1007/s40820-022-00906-5)
Supplement: Supplementary file 1 — Supplementary file1 (PDF 2343 KB) [file 40820_2022_906_MOESM1_ESM.pdf]

Supporting Information for

Ultrabroad Microwave Absorption Ability and Infrared Stealth Property of Nano-Micro CuS@rGO Lightweight Aerogels

Yue Wu[1], Yue Zhao[1], Ming Zhou[1], Shujuan Tan[1,*], Reza Peymanfar[2], Bagher Aslibeiki[3], Guangbin Ji[1,*]

[1] College of Materials Science and Technology, Nanjing University of Aeronautics and Astronautics, Nanjing 210016, P. R. China

[2] Department of Chemical Engineering, Energy Institute of Higher Education, Saveh, Iran

[3] Faculty of Physics, University of Tabriz, Tabriz, 51666-16471, Iran

*Corresponding authors. E-mail: gbji@nuaa.edu.cn (Guangbin Ji); tanshujuan@nuaa.edu.cn (Shujuan Tan)

Supplementary Tables and Figures

Table S1 Physical parameters of rC composite aerogels

Samples	Bottom radius (cm)	Height (cm)	Weight (g)	Density (g cm^{-3})
rC-1	0.92	2.27	0.0722	0.0110
rC-2	1.10	2.27	0.0980	0.0113
rC-3	0.91	2.31	0.0725	0.0121
rC-4	0.96	2.46	0.1087	0.0152
rC-5	0.81	2.28	0.0752	0.0160

Table S2 MA ability and IR emissivity of rC aerogels

Samples	RLmin (dB)	d (mm)	EAB (GHz)	d (mm)	Filler content (wt%)	IR emissivity	
						3~5 mm	8~14 mm
rC-1	-12.3	2.0	5.56	2.0	6	0.9858	0.9914
rC-2	-16.1	2.0	5.48	2.5	6	0.9412	0.9568
rC-3	-40.2	2.3	8.44	2.8	6	0.9062	0.9263
rC-4	-50.4	2.0	7.16	2.3	6	0.8680	0.9115
rC-5	-38.4	3.0	6.36	2.5	6	0.6442	0.8160

Table S3 Microwave absorption performance of RC aerogels

Samples	RL_{min} (dB)	d (mm)	EAB (GHz)	d (mm)	Filler content (wt%)
RC-1	-32.0	4.0	5.80	2.0	2
	-10.3	3.5	1.20	3.5	1
RC-2	-16.6	2.0	5.12	2.0	2
	-17.8	3.5	4.88	3.0	1
RC-3	-21.5	2.5	6.30	2.5	2
	-37.9	2.85	6.50	2.5	1
RC-4	-60.3	3.5	7.20	2.45	2
	-63.5	2.25	5.80	2.25	1
RC-5	-16.2	2.5	5.70	2.5	2
	-25.3	3.0	6.04	3.0	1

Table S4 Surface temperature of rC composite aerogels

T (°C) \ *t* (min)	0	5	10	15	20	25	30
rC-1	26.4	31.6	31.1	29.9	30.7	30.5	30.6
rC-2	26.6	31.4	31.5	31.7	30.7	30.0	30.3
rC-3	26.4	28.4	30.5	30.4	30.7	30.1	29.7
rC-4	26.6	27.1	27.4	26.6	26.8	26.8	26.8
rC-5	26.5	27.3	27.1	27.2	26.8	26.6	26.5

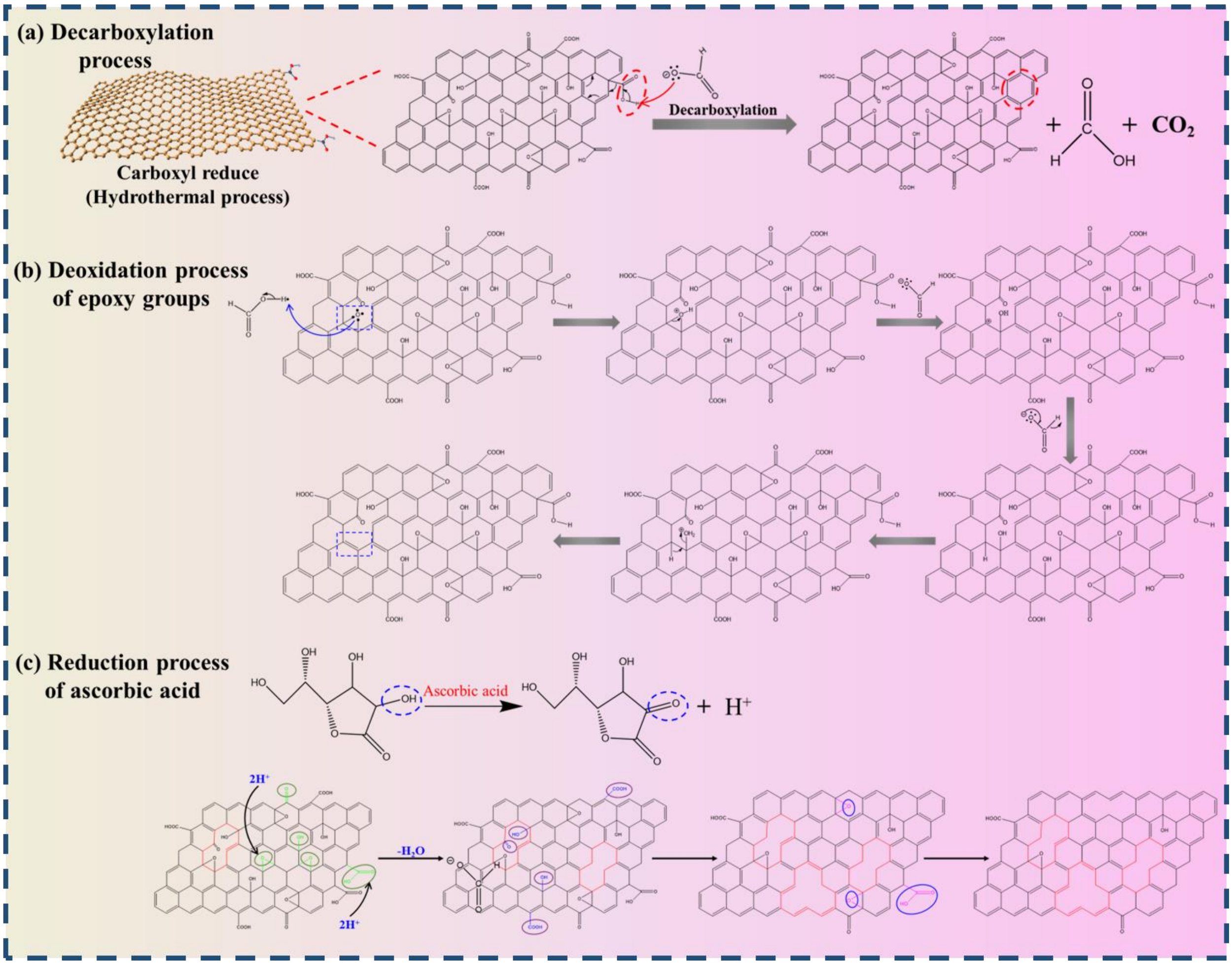

Fig. S1 Hydrothermal reduction mechanisms of **a** Deoxidation process of carboxyl groups and **b** deoxidation process of epoxy groups of rC composite aerogels. **c** Reduction process of ascorbic acid for RC composite aerogels

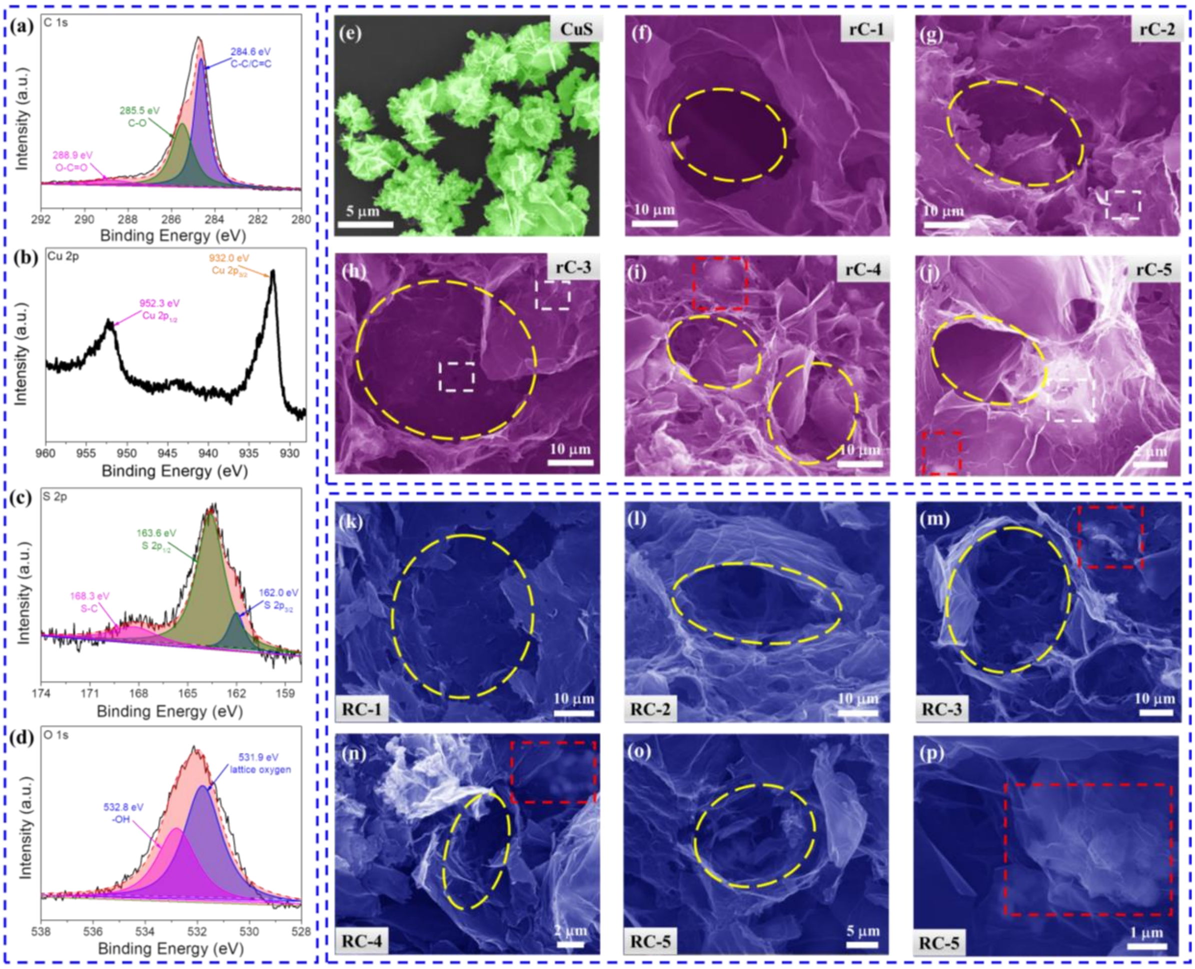

Fig. S2 XPS spectra of rC-4: **a** C 1s, **b** Cu 2p, **c** S 2p and **d** O 1s. SEM images of **e** CuS, **f** rC-1, **g** rC-2, **h** rC-3, **i** rC-4, and **j** rC-5. SEM images of **k** RC-1, **l** RC-2, **m** RC-3, **n** RC-4, **o, p** RC-5

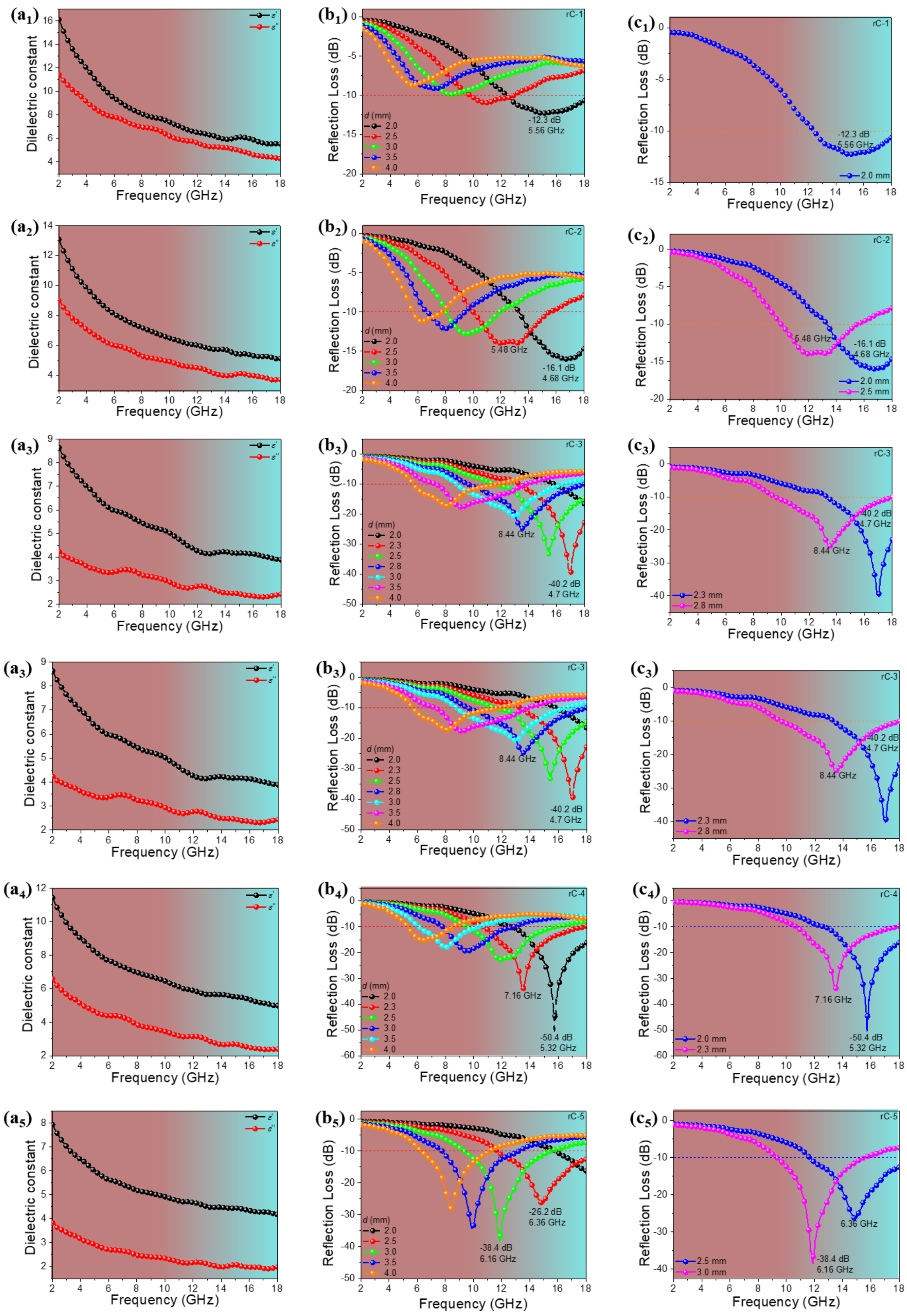

Fig. S3 a1-a5 Dielectric constant of ε' and ε'', **b1-b5** RL curves, **c1-c5** RL curves with minimum RL and broadest EAB of rC composite aerogels at specific thicknesses

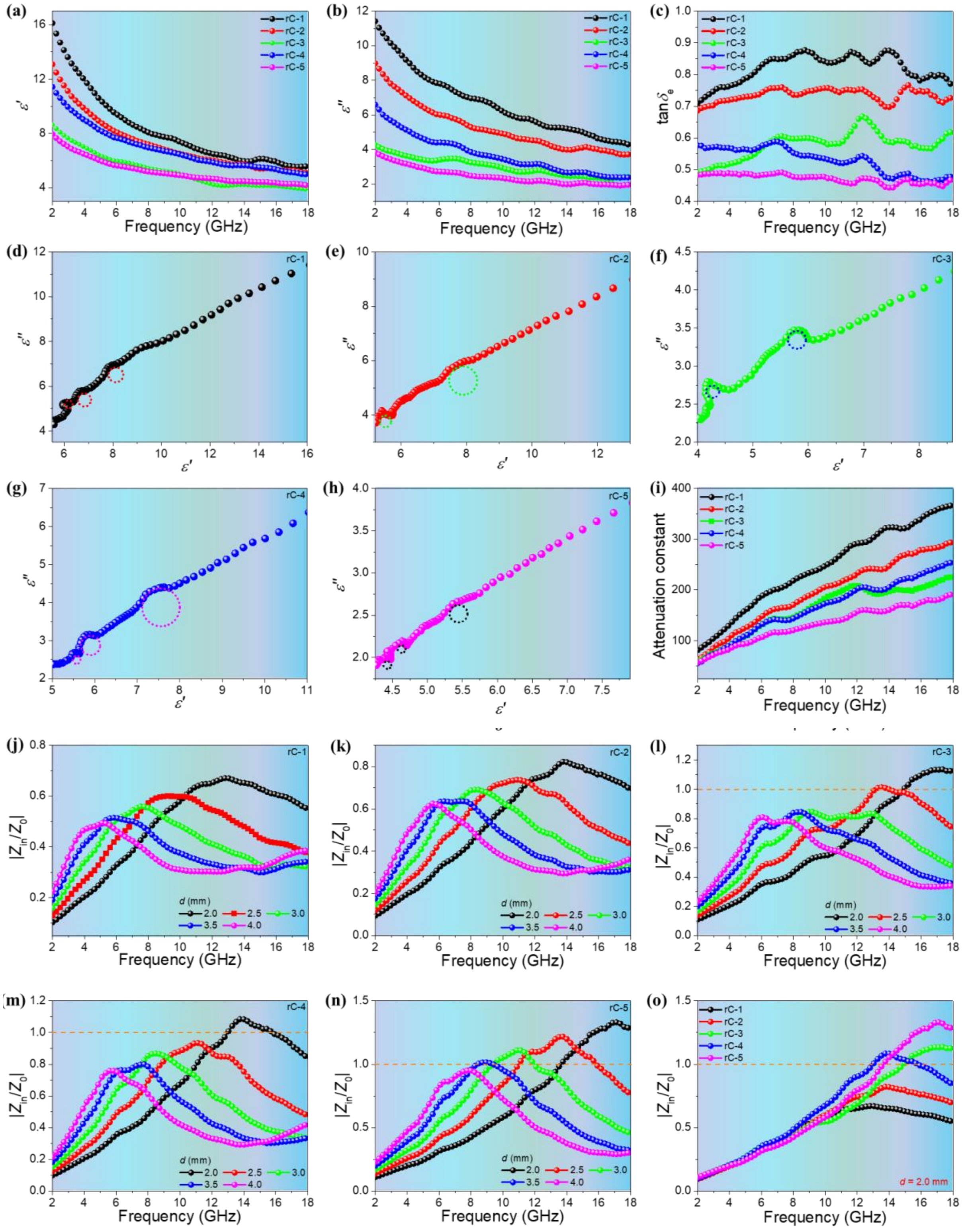

Fig. S4 a ε', **b** ε'', c $\tan\delta_e$, Cole-Cole curves of **d** rC-1, **e** rC-2, **f** rC-3, **g** rC-4 and **h** rC-5. **i** Attenuation constant of rC composite aerogels. Normalized input impedance $|Z_{in}/Z_0|$ of **j** rC-1, **k** rC-2, **l** rC-3, **m** rC-4 and **n** rC-5. **o** Normalized input impedance of rC composite aerogels at the thickness of 2.0 mm

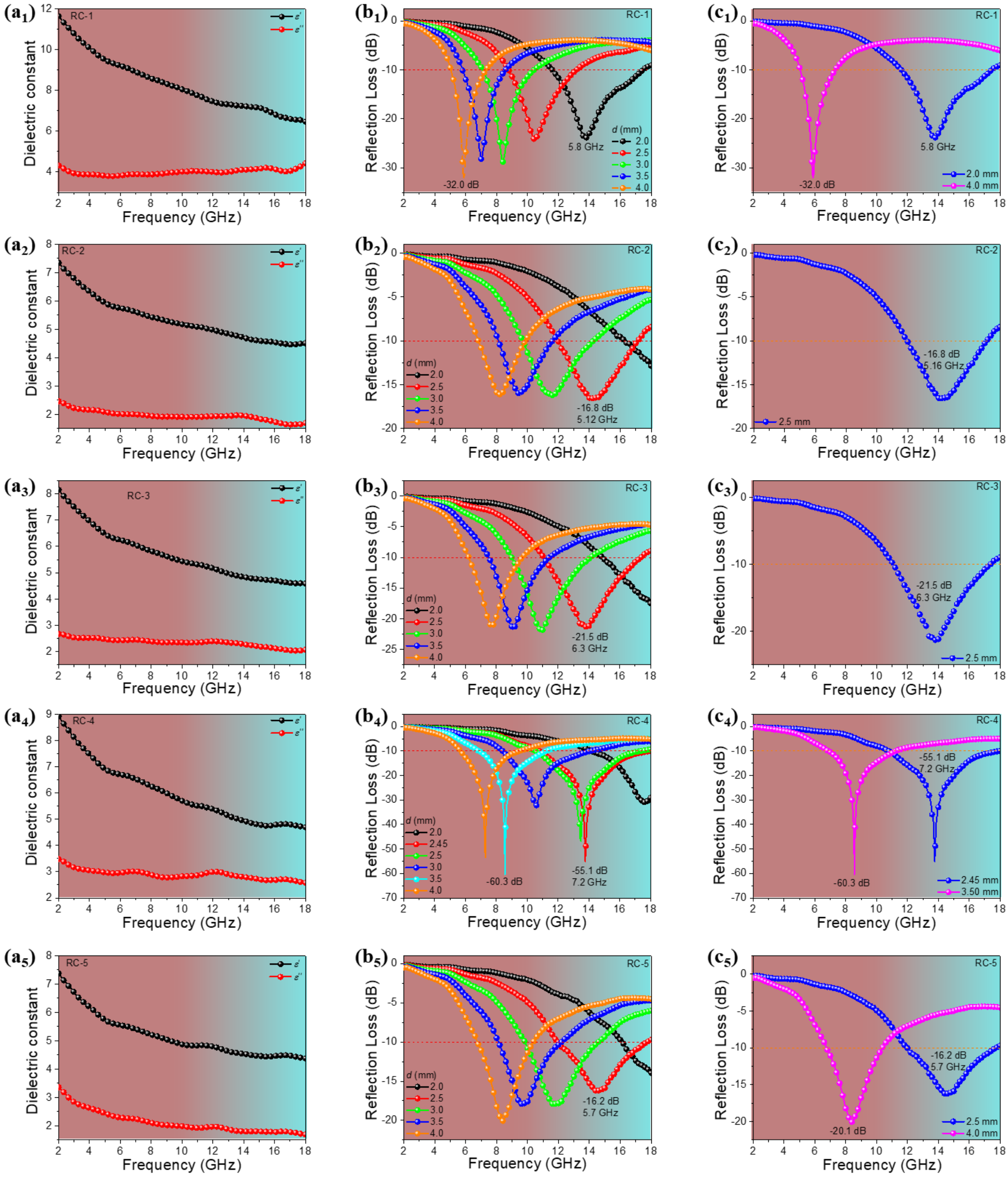

Fig. S5 a1-a5 Dielectric constant of ε' and ε'', **b1-b5** RL curves, **c1-c5** RL curves with minimum RL and broadest EAB of RC composite aerogels at specific thicknesses

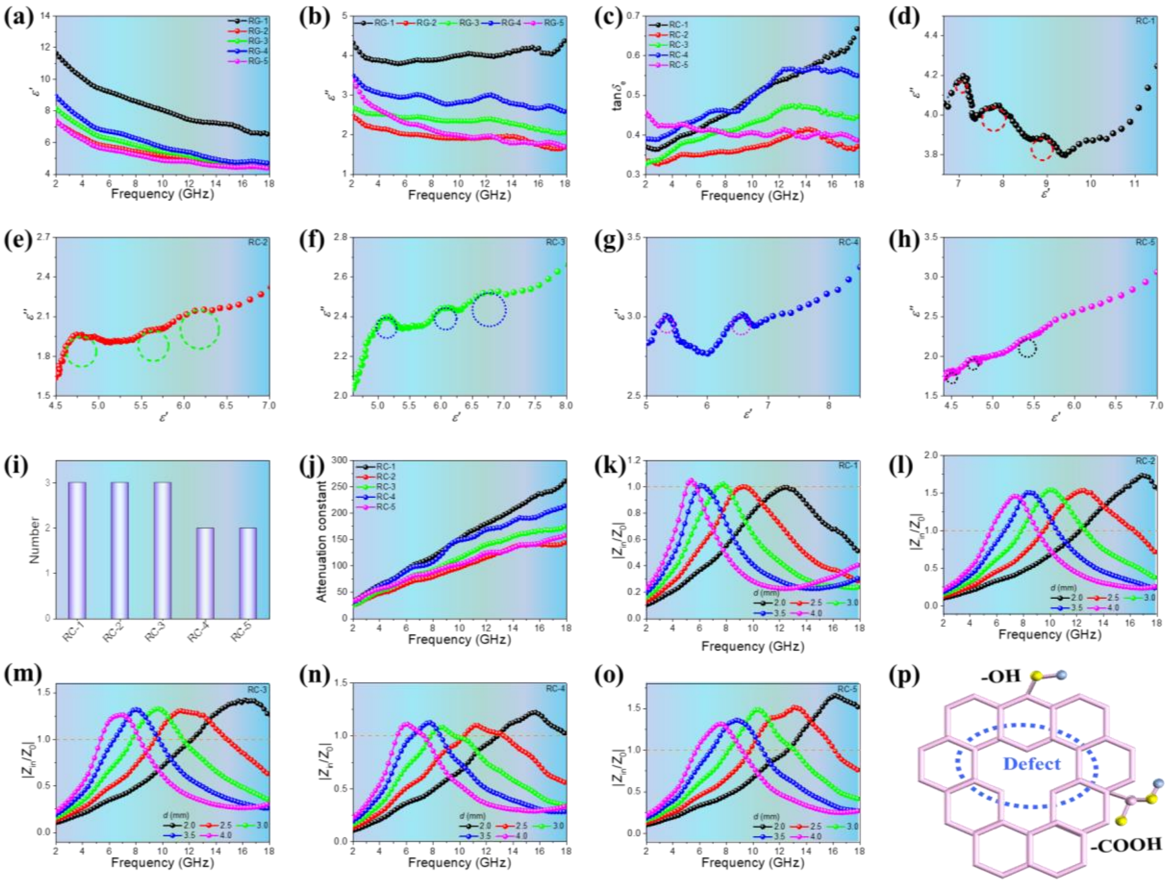

Fig. S6 a ε', **b** ε'', **c** tanδ_e of RC composite aerogels. Cole-Cole curves of **d** RC-1, **e** RC-2, **f** RC-3, **g** RC-4 and **h** RC-5. **i** Number of Cole-Cole semicircles of RC composite aerogels. **j** Attenuation constant of RC composite aerogels. Normalized input impedance $|Z_{in}/Z_0|$ of **k** RC-1, **l** RC-2, **m** RC-3, **n** RC-4 and **o** RC-5. **p** Defects and oxygen-containing functional group in RC composite aerogels

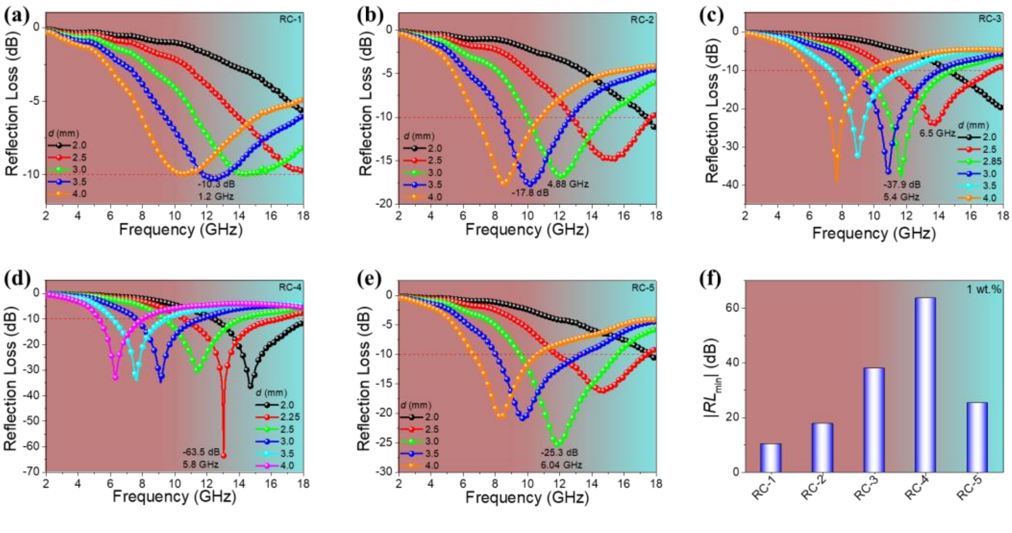

Fig. S7 RL curves of **a** RC-1, **b** RC-2, **c** RC-3, **d** RC-4 and **e** RC-5. **f** Histogram of $|RL_{min}|$ *vis* RC composite aerogels with the low filler content of 1 wt.%

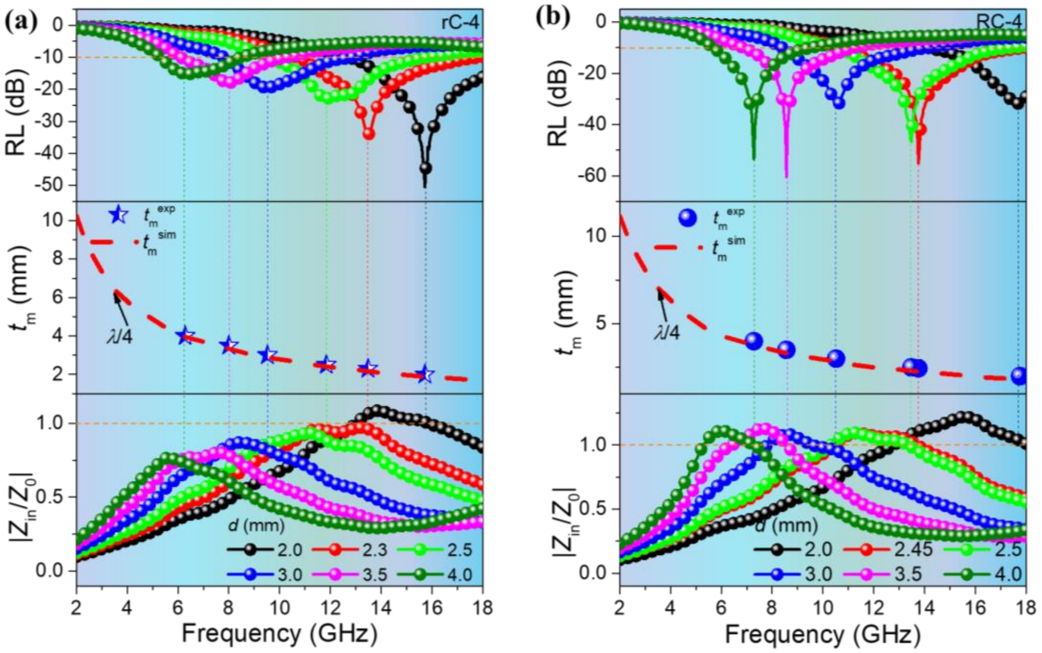

Fig. S8 RL, $\lambda/4$ and $|Z_{in}/Z_0|$ of **a** rC-4 and **b** RC-4

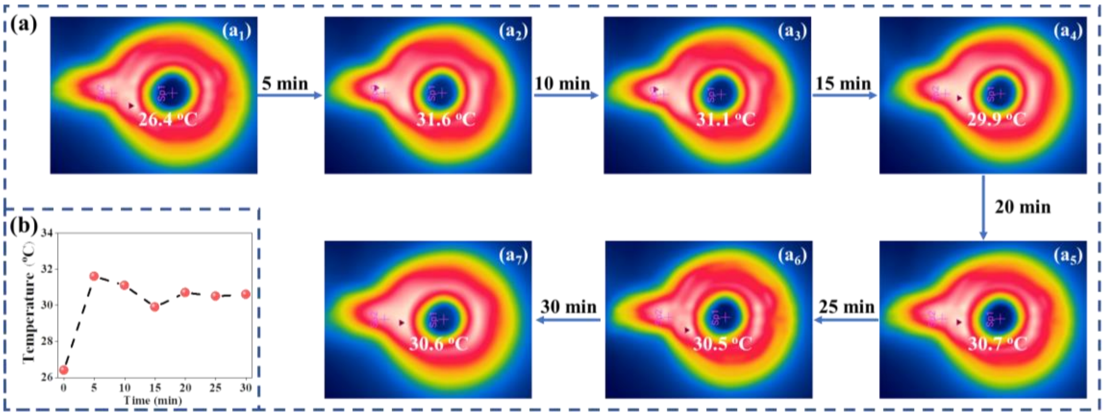

Fig. S9 **a** Thermal IR images of rC-1 at different heating times. **b** Surface temperature curve of rC-1

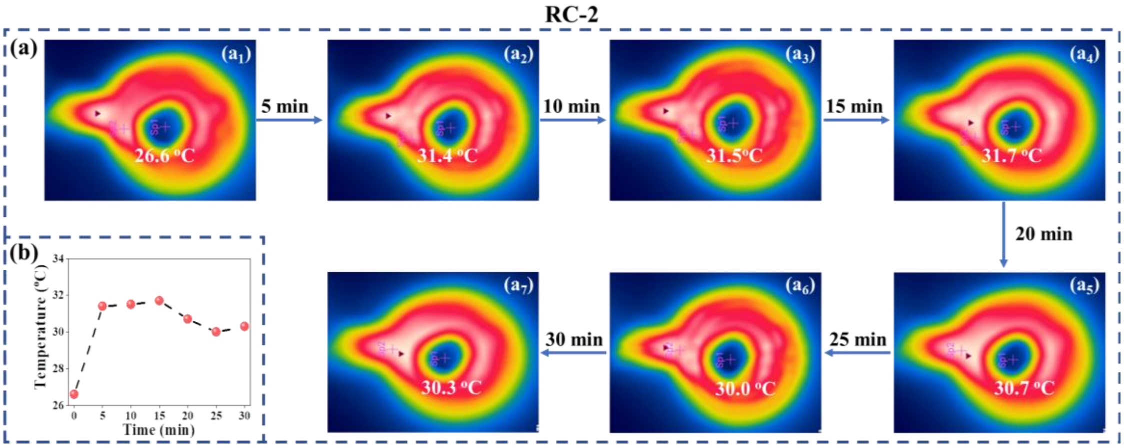

Fig. S10 **a** Thermal IR images of rC-2 at different heating times. **b** Surface temperature curve of rC-2

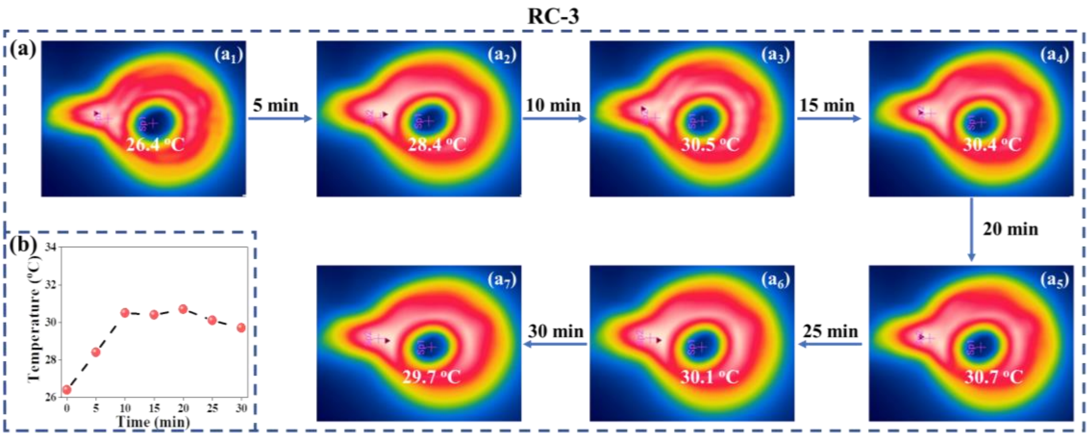

Fig. S11 a Thermal IR images of rC-3 at different heating times. **b** Surface temperature curve of rC-3

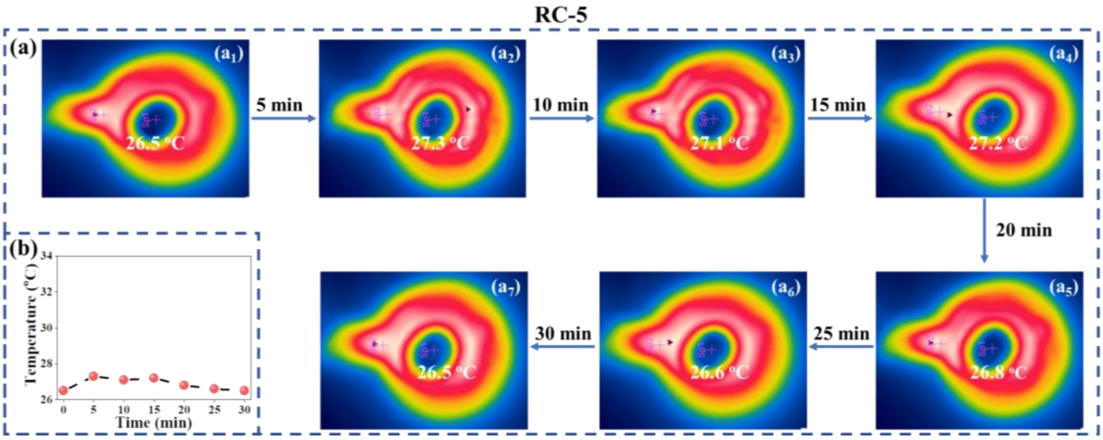

Fig. S12 a Thermal IR images of rC-5 at different heating times. **b** Surface temperature curve of rC-5

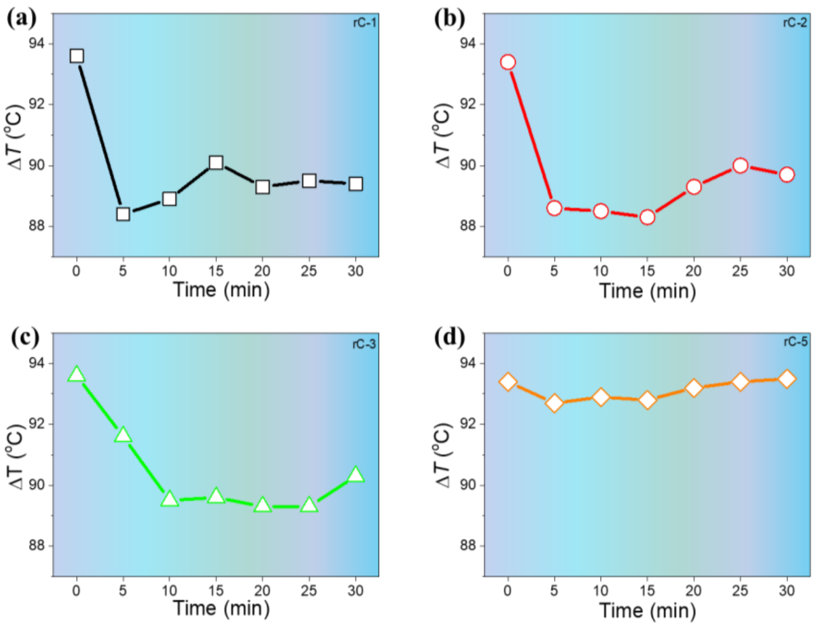

Fig. S13 Difference between heating temperature and surface temperature of **a** rC-1, b rC-2, c rC-3 and d rC-5
